# Intestinal decontamination with rifaximin attenuates LSEC dysfunction and liver fibrosis in mice

**DOI:** 10.1371/journal.pone.0340664

**Published:** 2026-01-23

**Authors:** Tingting Su, Sanchuan Lai, Hongtan Chen

**Affiliations:** 1 Department of Gastroenterology, The First Affiliated Hospital, Zhejiang University School of Medicine, Hangzhou, China; 2 Department of Gastroenterology, The Second Affiliated Hospital, Zhejiang University School of Medicine, Hangzhou, China; THe University of Texas in Austin, UNITED STATES OF AMERICA

## Abstract

**Background:**

The gut microbiome plays a pivotal role in the development and progression of liver disease. Liver sinusoidal endothelial cells (LSECs), as the first hepatic barrier exposed to blood from the portal circulation, may be influenced by gut-derived microbiota and their byproducts. This study aimed to investigate the interaction between gut microbiota and LSECs and to clarify how this interaction impacts the progression of liver cirrhosis.

**Methods:**

Liver cirrhosis was induced by carbon tetrachloride (CCl_4_) injection and bile duct ligation (BDL). CCl_4_ and BDL mice were administered rifaximin. The primary LSECs were isolated from mice and treated with LPS. 16S rRNA sequencing was conducted to examine changes in the gut microbiota of cirrhotic mice following rifaximin treatment.

**Results:**

Rifaximin attenuated liver fibrosis and LSEC dysfunction in CCl_4_ and BDL mice. Liver cirrhosis induced remarkable changes in the gut microbiome while rifaximin treatment could partially reverse these alterations. Serum lipopolysaccharides (LPS) level was elevated in cirrhotic mice, while reduced following rifaximin treatment. Furthermore, LPS treatment could induce LSEC dysfunction by inhibiting eNOS mRNA expression, which was attenuated by TLR4 inhibitor, indicating that TLR4 signaling was involved in LPS-induced LSEC dysfunction.

**Conclusions:**

Intestinal microbiota dysbiosis allows more LPS to enter the portal circulation, which may in turn exacerbate LSEC dysfunction and liver fibrosis. Intestinal decontamination with rifaximin improves LSEC function and alleviates liver fibrosis, a process linked to the reconstruction of the gut microbiome and a reduction in gut-derived LPS.

## Introduction

Liver cirrhosis is the end-stage of chronic liver disease (CLD). Regardless of its etiologies, chronic liver injury causes persistent inflammation and fibrosis, inducing collapse of liver structure and distortion of vascular architecture, finally leading to cirrhosis [1,2].

The gut microbiome may play a pivotal role in the development and progression of liver disease [3,4]. Gut microbial dysbiosis is commonly found in patients with CLD. Microbiota alterations occur in the early stages of CLD, particularly in alcoholic liver disease and nonalcoholic fatty liver disease (NAFLD) [5,6]. Importantly, microbiota profile alterations have been identified in cirrhotic patients, independent of cirrhosis etiology [7–9]. Gut microbial dysbiosis causes intestinal inflammation and gut barrier dysfunction, facilitating the translocation of pathogens and bacterial metabolites across the gut epithelial barrier into the portal circulation, a process known as bacterial translocation [3,10]. These translocated microbial products may worsen CLD and cirrhosis by promoting hepatocyte damage, inflammation, fibrosis, vascular resistance, and angiogenesis [2,11–14]. However, the interactions of liver cells with gut microbiota and its underlying mechanisms have not yet been clearly elucidated.

Liver sinusoidal endothelial cells (LSECs) play a crucial role in maintaining liver homeostasis and are actively involved in the pathogenesis of liver fibrosis/cirrhosis [15]. Healthy LSECs maintain specialized phenotype and are able to produce eNOS-derived NO, protecting the liver from fibrosis by preventing hepatic stellate cell (HSC) activation [16–18]. During liver injury, LSECs gradually lose their characteristic fenestrations and develop a basement membrane. This phenotypic transformation, known as sinusoidal capillarization, is accompanied by a functional shift from an anti-fibrotic to a pro-fibrotic state [15,19]. Decreased production of eNOS-derived NO is a fundamental characteristic of LSEC dysfunction [15,17]. In addition, dysfunctional LSECs exhibit significant alterations in their angiocrine profile, accompanied by elevated secretion of proinflammatory mediators, vasoconstrictors, and profibrotic factors, resulting in dysregulated sinusoidal homeostasis and aggravated liver fibrosis [19].

The liver receives 70% of the blood supply from the intestinal portal circulation [20]. LSECs, which line the hepatic sinusoids, act as the primary barrier against gut-derived microbiota and their metabolites entering through the portal circulation [21]. Conversely, these microbial components can also influence LSECs, although the underlying mechanisms were poorly explored. Formes et al. conducted a comparative whole-transcriptome analysis, revealing that the LSEC transcriptome of germ-free mice significantly differed from that of conventionally raised mice, including notable alterations in sphingolipid metabolism-related gene expression [22]. Another study demonstrated a positive correlation between LSEC fenestrae porosity and diameter with the relative abundance of Firmicutes in the gut microbiota, while an inverse correlation was found with Bacteroidetes abundance [23]. Furthermore, the elevated circulating lipopolysaccharide (LPS) levels have been linked to fenestrae loss in LSECs [24]. However, these studies are primarily based on correlation analyses and transcriptomic studies. Further mechanistic investigations are necessary to clarify how gut microbiota regulate LSEC function.

Considering the gatekeeper roles of LSECs in liver fibrogenesis and immunity, it was hypothesized that gut microbial dysbiosis may aggravate LSEC dysfunction, promoting liver fibrosis. Hence, the non-absorbable antibiotic rifaximin was administered to cirrhotic mice to assess the effects of microbial changes on LSEC dysfunction and liver fibrosis/cirrhosis.

## Materials and methods

### Animal experiments

Liver fibrosis/cirrhosis was induced by carbon tetrachloride (CCl_4_) injection for 8 weeks [11] or bile duct ligation (BDL) for 3 weeks, as previously described [25]. For the CCl4 model, 6-week-old male C57BL/6J mice were injected intraperitoneally with CCl_4_ (Sigma-Aldrich, 5 mL/kg body weight, diluted in olive oil at a ratio of 1:9) twice a week. For the BDL model, 8-week-old male C57BL/6J mice were used. The BDL surgery was performed under sodium pentobarbital anesthesia. Age-matched mice and sham-operated mice were utilized as controls for the CCl4 and BDL models, respectively. Mice were administered daily oral gavage of rifaximin (100 mg/kg body weight) [26–28] or PBS, starting from the first day of CCl₄ injection or bile duct ligation (BDL) surgery until sacrifice. There were no mice died in CCl_4_ model and 1–2 mice died per group in BDL model. We euthanized the mice by neck dissection under anesthesia with inhalation of isoflurane. And all efforts have been made to minimize suffering. The researchers were train in animal care and handling by Zhejiang University Laboratory Animal Center. The mice were purchased from GemPharmatech Co.(Jiangsu China). All the animal experiments were approved by the Animal Care and Use Committee of the First Affiliated Hospital, Zhejiang University School of Medicine (Hangzhou, China).

### Primary LSEC isolation and culture

The primary LSECs were isolated from control, CCl_4_-treated, and BDL mice, as previously described with some modifications [29]. Firstly, liver nonparenchymal cells (NPCs) were isolated using a liver dissociation kit (Miltenyi Biotec) and processed with the gentleMACs^TM^Octo Dissociator (Miltenyi Biotec) according to the manufacturer’s instructions. The NPC suspension was subsequently subjected to a density gradient centrifugation using Percoll (Sigma-Aldrich, Lot p4936, 25/50%) at 900 x g for 20 min at 4^o^C. The cell fraction at the 25/50% interface was collected and incubated in plates for 10 min to remove Kupffer cells. The isolated LSECs were resuspended in Microvascular Endothelial Growth Media-2 (EGM™-2MV Medium, Lonza, CC-3202) and seeded onto collagen-coated plates. Cells were cultured at 37°C in a humidified atmosphere with 5% CO₂ overnight and subsequently used for immunofluorescence staining or LPS (Sigma-Aldrich, L4391) treatment. To inhibit TLR4 signaling, LSECs were preincubated with TAK-242 (Resatorvid; MCE) for 2 h prior to LPS treatment.

### H&E and sirius red staining

Paraffin-embedded liver tissue slides were deparaffinized with xylene and rehydrated through a graded ethanol series. For H&E staining, tissue slides were incubated in hematoxylin solution for 1 min, followed by placement into eosin solution for 30 sec. For Sirius Red staining, slides were immersed in 0.1% Sirius Red solution (0.5 g Sirius Red F3B dissolved in 500 mL saturated aqueous picric acid solution) for 1 h. The slides were then washed twice in acidified water, with each wash consisting of 10 dips. After dehydration, the slides were mounted and observed under an ECLIPSE CIL microscope (Nikon, Tokyo, Japan).

### Immunohistochemistry

Paraffin-embedded liver tissue slides were deparaffinized with xylene and rehydrated through a graded ethanol series. Heat-induced antigen retrieval was performed using HIER citrate buffer (pH 6.0) under high pressure for 3 min. Endogenous peroxidase activity was blocked with 3% H₂O₂, and nonspecific binding was inhibited with 5% bovine serum albumin (BSA). Slides were incubated with primary antibodies overnight at 4°C, followed by incubation with a labeled secondary antibody at 37°C for 30 min. Chromogenic detection was carried out using DAB, and hematoxylin was applied for counterstaining. After dehydration, clearing, and mounting, slides were examined under a Nikon ECLIPSE CIL microscope. The primary antibodies used were rabbit anti-COL3A (1:1000, #22734–1-AP; Proteintech, Rosemont, IL, USA), rabbit anti-CD31 (1:2500, #ab281583; Abcam, Cambridge, UK), rabbit anti-LYVE1 (1:2000, #ab314241; Abcam).

### Cell immunofluorescence assay

LSECs seeded on cover glasses were fixed with 4% paraformaldehyde (PFA) for 20 min, permeabilized with 0.3% Triton X-100 for 10 min, blocked with 5% donkey serum for 60 min, and incubated with primary antibodies in a humidified chamber overnight. Rabbit anti-LYVE1 (1:100, #ab14917; Abcam, rat anti-CD31 (1:50, #sc-18916; Santa Cruz Biotechnology, Dallas, TX, USA), and rabbit anti-NFκB (1:400, #8242; CST, Boston, UK) were used as primary antibodies.

### Western blot assay

Total proteins were extracted from primary LSECs or liver tissues using RIPA lysis buffer and quantified with the BCA Protein Assay Kit (Vazyme, Nanjing, China). Proteins were electrophoresed through 10% sodium dodecyl sulfate (SDS)-polyacrylamide gels and then transferred onto polyvinylidene difluoride (PVDF) membranes. The membranes were blocked with 5% BSA for 2 h and incubated with primary antibodies at 4°C overnight. The following primary antibodies were used: rabbit anti-a-SMA (1:10000, #ab124964; Abcam), rabbit anti-eNOS (1:1000, #ab199956; Abcam), rabbit anti-p-eNOS (1:1000, #ab215717; Abcam), rabbit anti-TLR4 (1:1000, #A5258; ABclonal, Wuhan, China), rabbit anti-MyD88 (1:8000, #A22600; ABclonal), rabbit anti-NFκB (1:1000, #8242; CST), rabbit anti-p-NFκB (1:1000, #3033; CST). Membranes were subsequently incubated with horseradish peroxidase (HRP)-labeled secondary antibodies at room temperature for 2 h on the following day. Protein signals were detected using an ECL kit (FDbio Science, Hangzhou, China). GAPDH was used as a reference gene.

### Real-time quantitative reverse transcription polymerase chain reaction (RT-qPCR)

Total RNAs were extracted from primary LSECs using Trizol reagent (Vazyme), which were then reversely transcribed to cDNA using the HiScript II Q RT SuperMi kit (Vazyme,Nanjing,China). The RT-qPCR was performed using the ROCHE LightCycler® 480 system (Rotor-Gene 6000 software, Sydney, Australia). Each reaction was conducted in triplicate in a 10 µL reaction volume containing ChamQ SYBR qPCR Master Mix (Vazyme), primers, and template DNA. β-actin served as the internal reference gene. The primer sequences were provided by PrimerBank (https://pga.mgh.harvard.edu/primerbank/). The following primers were used in RT-qPCR:

eNOS-F, 5’-TGTGACCCTCACCGCTACAA-3’,
eNOS-R,5’-GCACAATCCAGGCCCAATC-3’;
β-actin-F, 5’-GGCTGTATTCCCCTCCATCG-3’,
β-actin-R, 5’-CCAGTTGGTAACAATGCCATGT-3’;
α-SMA -F, 5’-TGGCACCACTCTTTCTATAACG-3,
α-SMA -R 5’-GGTCATTTTCTCCCGGTTGG-3;
LYVE1-F, 5’- CAGCACACTAGCCTGGTGTTA-3,
LYVE1-R 5’- CGCCCATGATTCTGCATGTAGA-3;
VWF-F, 5’- CTTCTGTACGCCTCAGCTATG-3,
VWF-R 5’- GCCGTTGTAATTCCCACACAAG-3.

### The enzyme-linked immunosorbent assay (ELISA)

Serum LPS level was measured using a Mouse LPS ELISA kit (ELK Biotechnology, Denver, CO, USA), and Procollagen III N-Terminal Propeptide (PIIINP) level was quantified using an ELISA kit for PIIINP (#SEA573Mu, Cloud-Clone Corp), following the manufacturer’s instructions. Briefly, standards or murine serum samples were added to microtiter plate wells pre-coated with LPS or PIIINP antibodies. Biotin-conjugated LPS or PIIINP antibodies were then added, followed by avidin conjugated to HRP and TMB substrate solution. The enzyme-substrate reaction was terminated with sulfuric acid solution, and the color change was measured spectrophotometrically at 450 nm. LPS and PIIINP levels were determined by comparing the optical density (OD) values of the samples to the standard curve.

### Nitric oxide (NO) detection

The culture media of LSECs were collected, and the NO concentration was detected using a Total Nitric Oxide Assay kit (Beyotime, Shanghai, China) according to the manufacturer’s instructions.

### Hydroxyproline (HYP) content assay

The hydroxyproline content in liver tissues was quantified using a Hydroxyproline Content Assay Kit (Boxbio, Lot AKAM017M) according to the manufacturer’s instructions. Briefly, approximately 0.2g of liver tissue was hydrolyzed in 2 mL of hydrochloric acid (6 mol/L) at 110°C for 3 hours. After centrifugation (12000g, 20 min), the supernatant was collected and the pH was adjusted to 6.0–8.0 before being diluted to a final volume of 4 mL with distilled water. The processed sample was then mixed with the assay reagents, and the absorbance was measured at 560 nm using a microplate reader. A standard curve was generated with provided hydroxyproline standards, and the hydroxyproline content in the samples were calculated and expressed as micrograms per gram of wet liver tissue (μg/g).

### 16S rRNA sequencing and data processing

Fecal murine samples were collected prior to sacrifice. Total microbial DNA was extracted from the fecal samples using the PF Mag-Bind Stool DNA kit (Omega Bio-tek, GA, USA) according to the manufacturer’s protocol. The V3-V4 region of the 16S rRNA gene was amplified by PCR using the primers 338F (5’-ACTCCTACGGGAGGCAGCAG-3’) and 806R (5’-GGACTACHVGGGTWTCTAAT-3’) [30]. After purification, the PCR products were paired-end sequenced on an Illumina PE300 platform (Illumina, San Diego, CA, USA) according to the protocols provided by Majorbio Bio-Pharm Technology Co. Ltd. (Shanghai, China). Raw FASTQ files were processed using FASTP 0.19.6 [31] and merged using FLASH 1.2.11 [32]. The optimized sequences were clustered into operational taxonomic units (OTUs) using UPARSE 11 [33] with a 97% sequence similarity threshold. Taxonomy for each OTU was determined using RDP Classifier 2.13 [34] based on the 16S rRNA gene database(e.g., Silva v138).

### Statistical analysis

Bioinformatic analysis of the 16S rRNA sequence data was performed using the Majorbio Cloud platform (https://cloud.majorbio.com). Alpha diversity indices, including the Ace index and Chao1, were calculated using Mothur 1.30.2 [35] Beta diversity in different samples was analyzed by principal coordinate analysis (PCoA) and non-metric multidimensional scaling analysis (NMDS) through Vegan 2.4.3 package. One-way analysis of variance (ANOVA) was performed to identify the significantly different abundant genera or species of bacteria between the two groups (*P* < 0.05).

Experimental results were evaluated using unpaired Student’s t-test for two groups and one-way ANOVA for multiple groups followed by Tukey’s post-hoc test for multiple comparisons. Two-tailed *p-*values less than 0.05 were considered statistically significant. The statistical analysis was conducted using GraphPad Prism 9.0 software (GraphPad Software Inc., San Diego, CA, USA).

## Results

### Rifaximin attenuated liver fibrosis in mice

**Fig 1:**
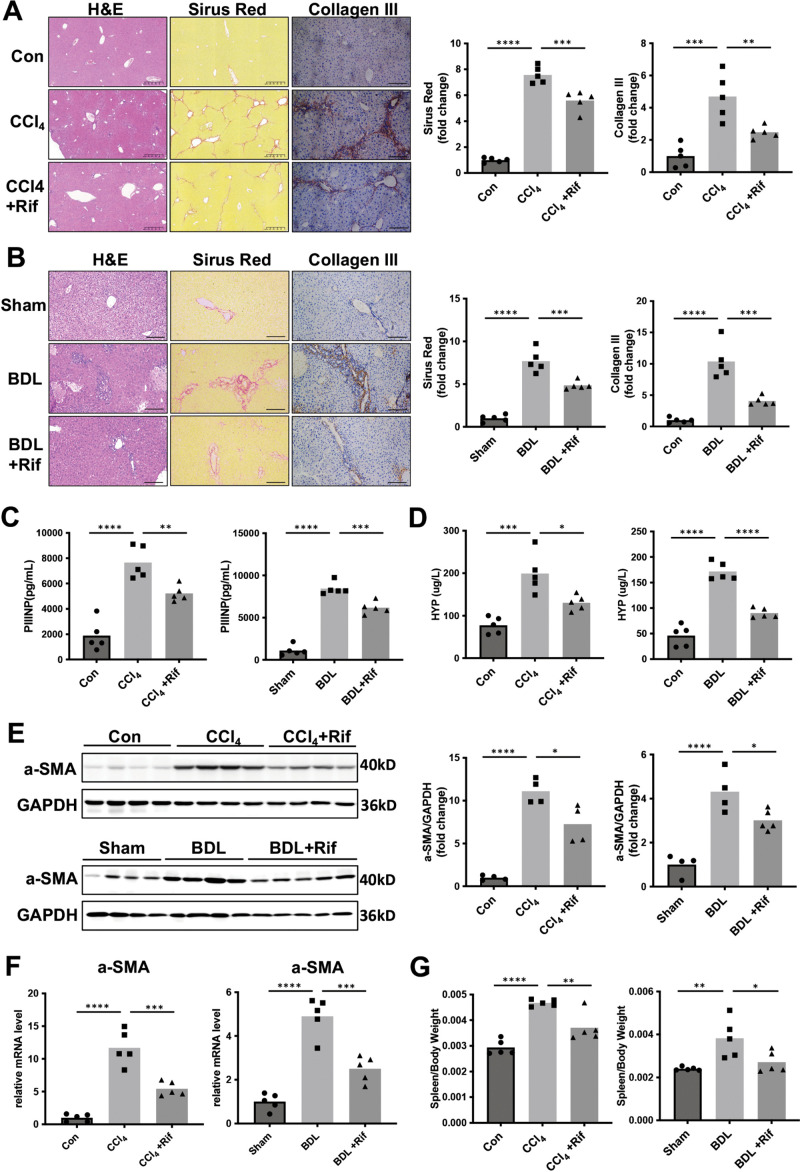
Rifaximin attenuated liver fibrosis. **A**-**B.** (left) H&E and staining of Sirius Red, Collagen III in liver tissue in CCl_4_ (A) and BDL mice (B) treated with or without rifaximin. (right) Quantification of positive area of staining using ImageJ (n = 5 mice per group). Scale bar: 400 μm. **C.** Serum PIIINP concentration in CCl_4_ (left) and BDL (right) mice treated with or without rifaximin, as measured by ELISA (n = 5 mice per group). **D.** Hydroxyproline content in liver tissues from CCl_4_ and BDL mice treated with or without rifaximin (n = 5 mice per group). **E.** Western blotting images and quantification of α-SMA in liver from CCl_4_ and BDL mice treated with or without rifaximin. GAPDH was used as a reference protein. (n = 4-5 mice per group). **F.** The relative mRNA expression of α-SMA in liver tissue from CCl_4_ and BDL mice treated with or without rifaximin (n = 5 mice per group). **G.** Spleen/body weight ratio in CCl_4_ (left) and BDL (right) mice treated with or without rifaximin (n = 5 mice per group). *P < 0.05, **P < 0.01, ***P < 0.001, ****P < 0.0001 (one-way ANOVA with Tukey’s post-hoc test). H&E, hematoxylin and eosin; PIIINP, procollagen III N-terminal propeptide; ELISA, the enzyme-linked immunosorbent assay; HYP, hydroxyproline.

To elucidate the potential impact of gut microbial alterations on the progression of liver fibrosis, liver fibrosis/cirrhosis was induced in murine models using CCl_4_ and BDL, followed by rifaximin gavage. Sirius red staining revealed a significant attenuation of liver fibrosis in both CCl_4_ and BDL models post-rifaximin treatment (Figs 1A, B). Moreover, rifaximin administration led to a significant reduction in the deposition of collagen III in the cirrhotic liver, as evidenced by immunohistochemical staining (Figs 1A, B). Correspondingly, serum levels of III N-terminal propeptide (PIIINP) were diminished in cirrhotic mice following rifaximin treatment (Fig 1C). Additionally, hydroxyproline (HYP) content assay in liver tissues confirmed a significant decrease in collagen deposition upon rifaximin treatment (Fig 1D). And the increased expression level of α-SMA in cirrhotic livers was also attenuated after rifaximin gavage (Fig 1E, F). The spleen-to-body weight ratio, which was elevated in both CCl4 and BDL models, also exhibited a remarkable decrease upon rifaximin treatment (Fig 1G), suggesting that rifaximin may mitigate portal hypertension.

### Rifaximin alleviated LSEC dysfunction in mice

**Fig 2:**
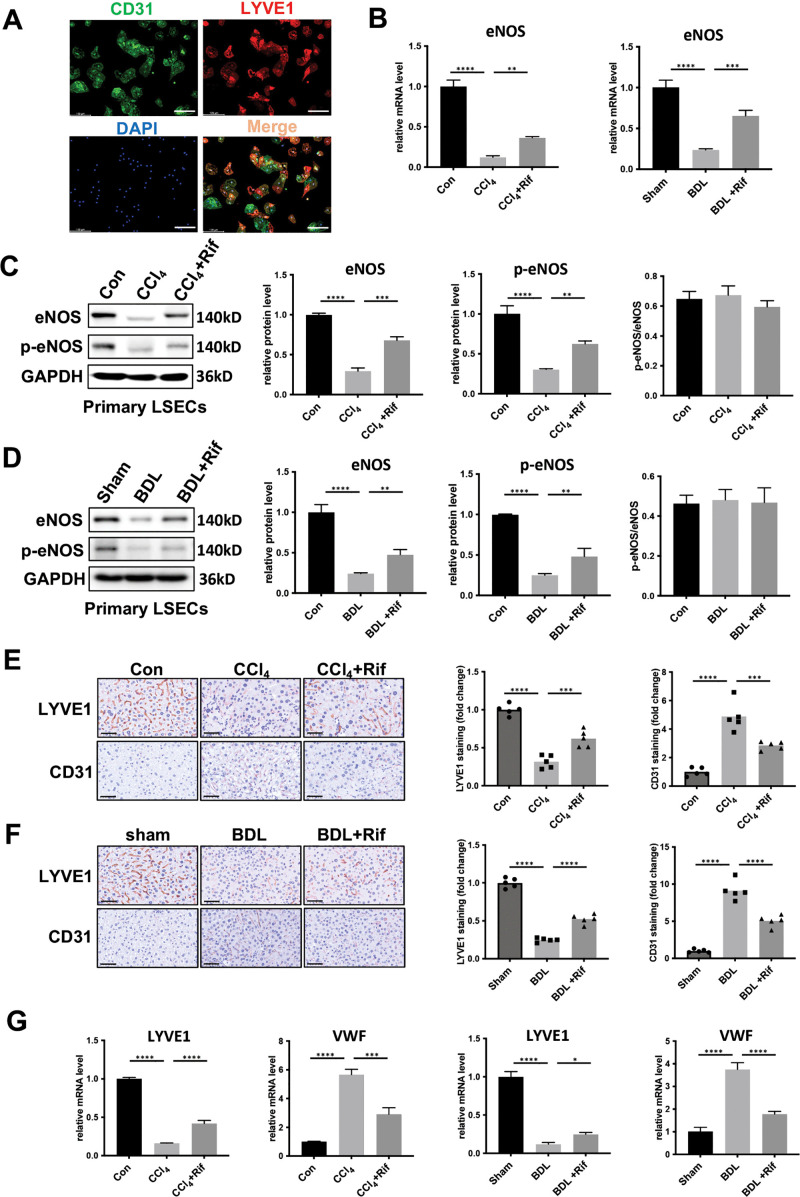
Rifaximin alleviated LSEC dysfunction. **A.** Immunocytochemistry staining of CD31 and LYVE-1 in murine primary LSECs. Scale bar: 100 μm. **B.** The relative mRNA expression of eNOS in primary LSECs isolated from control (sham), CCl_4_ (BDL), and rifaximin treated CCl_4_ (BDL) mice. **C-D.** Western blotting images and quantification of eNOS and p-eNOS in primary LSECs isolated from CCl_4_ (C) and BDL mice(D). GAPDH was used as a reference protein. **E-F.** (left)The staining of LYVE1 and CD31 in liver tissue in CCl_4_ (E) and BDL mice(F) treated with or without rifaximin. (right) Quantification of positive area of staining using ImageJ (n = 5 mice per group). Scale bar: 50 μm. **G.** The relative mRNA expression of LYVE1 and VWF in primary LSECs isolated from control (sham), CCl_4_ (BDL), and rifaximin treated CCl_4_ (BDL) mice. All data are presented as mean ± SD, *P < 0.05, **P < 0.01, ***P < 0.001, ****P < 0.0001 (one-way ANOVA with Tukey’s post-hoc test). LSECs, liver sinusoidal endothelial cells.

Given that LSECs form the primary interface between the liver and gut-derived microbial metabolites from the portal circulation, it was hypothesized that LSECs could be involved in the mitigation of liver fibrosis by rifaximin. Primary LSECs were isolated from murine models, and their identity was confirmed through immunofluorescence, which demonstrated co-localization of CD31 and LYVE1, key markers for LSECs (Fig 2A). The immunofluorescence analysis revealed variability in the expression levels of LYVE1 among LSECs, being consistent with previous studies. It was unveiled that the mRNA expression level of eNOS was significantly downregulated in LSECs isolated from cirrhotic livers compared with controls, accompanying by a notable upregulation after rifaximin treatment in both CCl_4_ and BDL models (Fig 2B). Similarly, western blot assay revealed that both eNOS and phosphorylated eNOS (p-eNOS) levels were significantly elevated in LSECs isolated from CCl_4_ and BDL mice following rifaximin treatment (Figs 2C, D), suggesting that rifaximin could partially mitigate LSEC dysfunction in cirrhotic livers. However, the ratio of p-eNOS to eNOS remained unaltered, indicating that rifaximin could modulate eNOS expression level without influencing its phosphorylation status (Figs 2C, D). Moreover, we also find rifaximin treatment upregulated sinusoidal endothelial cell-specific marker LYVE1, while downregulated continuous endothelial markers CD31 and VWF by immunohistochemical staining of liver tissue or RT-qPCR analysis of primary LSECs from both CCl_4_ and BDL mice (Figs 2E-G), demonstrating rifaximin is beneficial to maintain LSEC phenotype.

### Rifaximin induced alterations in the gut microbiota of mice

**Fig 3:**
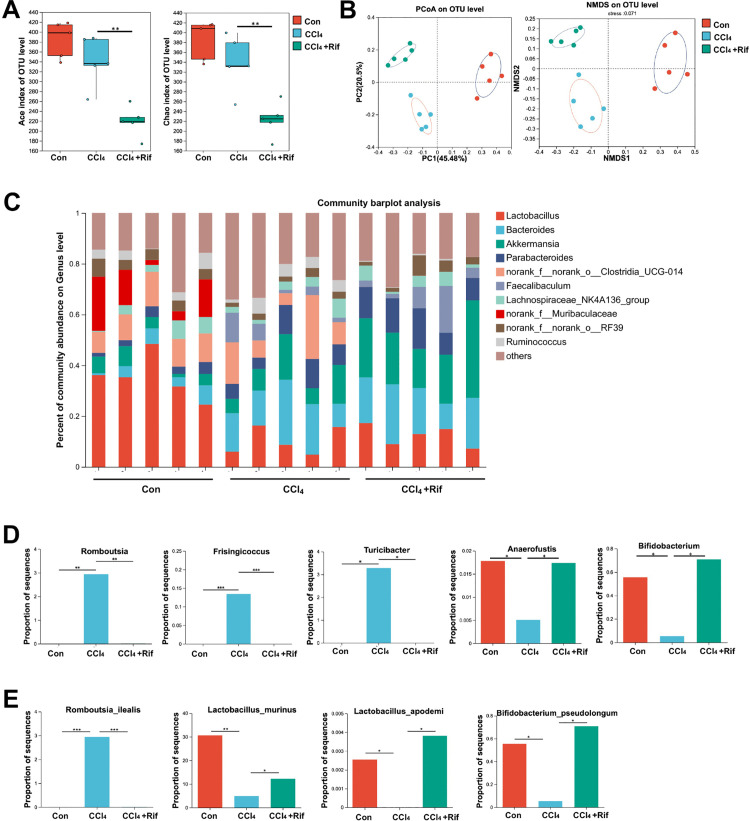
Rifaximin induced alterations in the gut microbiota of mice. **A.** Richness of the gut microbiota in mice, assessed using the Ace and Chao indices. (n = 5 mice per group). **B.** The β-diversity of gut microbiota in control and CCl_4_ mice treated with or without rifaximin, as indicated by PCoA and NMDS analysis. (n = 5 mice per group). **C.** The component of gut microbiota at the genus level in control and CCl_4_ mice treated with or without rifaximin. (n = 5 mice per group). **D-E.** Significant differences in the proportion of gut microbiota at the genus level (D) and species level (E) among the three groups. (n = 5 mice per group). *P < 0.05, **P < 0.01, ***P < 0.001, ****P < 0.0001 (one-way ANOVA with Tukey’s post-hoc test).

To figure out the alterations in gut microbiota in cirrhotic mice and the modulatory effects of rifaximin, fecal samples were collected from control, CCl_4_, and CCl_4_ + rifaximin-treated mice and subjected to 16S rRNA sequencing. The sequencing depth and rarefaction curves were demonstrated in S1 Fig. Notably, CCl_4_ mice exhibited a significant reduction in gut microbiota richness versus the control group, and rifaximin treatment further exacerbated this reduction, as assessed by the Ace and Chao diversity indices (Fig 3A). β-diversity analysis, including PCoA and NMDS, revealed distinct clustering of the samples based on experimental group, suggesting that liver cirrhosis induced remarkable alterations in the gut microbiome, which were further modified by rifaximin treatment (Fig 3B). At the genus level, the composition of the gut microbiota was markedly altered, as depicted in the bar plot (Fig 3C). Notably, genera, such as *Romboutsia*, *Frisingicoccus*, and *Turicibacter*, which were significantly elevated in cirrhotic mice, demonstrated a notable reduction following rifaximin treatment. Conversely, *Bifidobacterium* and *Anaerofustis*, which were depleted in cirrhotic mice, exhibited significant reconstitution following rifaximin administration (Fig 3D). At the species level, *Romboutsia ilealis*, which was overrepresented in the cirrhotic mice, was dramatically reduced after rifaximin treatment. Additionally, probiotic species, such as *Lactobacillus murinus*, *Lactobacillus apodemi*, and *Bifidobacterium pseudolongum*, which were diminished in the cirrhotic state, were notably re-induced by rifaximin (Fig 3E). These data suggest that rifaximin exerts a significant influence on the gut microbiota composition, partially reversing the dysbiosis associated with liver cirrhosis.

### LPS induced LSEC dysfunction by inhibiting eNOS mRNA expression

**Fig 4:**
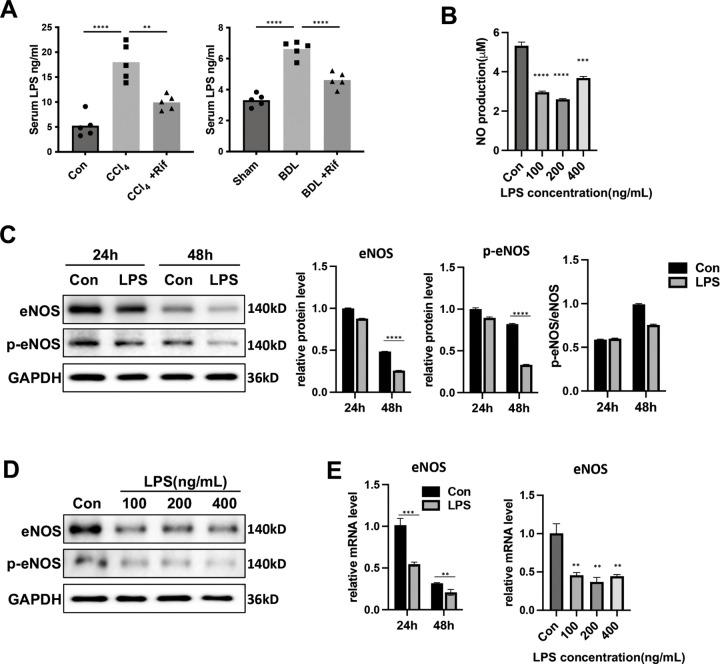
LPS induced LSEC dysfunction by inhibiting eNOS mRNA expression. **A.** Serum LPS concentration in CCl_4_ and BDL mice treated with or without rifaximin, as measured by ELISA (n = 5 mice per group). **B.** NO concentration in the culture medium of LSECs treated with LPS in different concentrations (100, 200, and 400 ng/mL). **C.** Western blotting images and quantification of eNOS and p-eNOS in primary LSECs treated with LPS (100 ng/mL) for 24h or 48h. GAPDH was used as a reference protein. **D.** Western blotting images of eNOS and p-eNOS in primary LSECs treated with LPS at different concentrations (100, 200, and 400 ng/mL) for 48h. GAPDH was used as a reference protein. **E.** The mRNA expression of eNOS in primary LSECs treated with LPS at different time points (24h or 48h) or at different concentrations (100, 200, and 400 ng/mL). All data are presented as mean ± SD, *P < 0.05, **P < 0.01, ***P < 0.001, ****P < 0.0001 (one-way ANOVA with Tukey’s post-hoc test in A, B, E(right); unpaired Student’s t-test in C,E(left)). NO, nitric oxide; LSECs, liver sinusoidal endothelial cells; LPS, lipopolysaccharide; ELISA, the enzyme-linked immunosorbent assay.

LPS, a potent product derived from Gram-negative bacteria, has the potential to enter the portal circulation via the gut and interact with LSECs under cirrhotic conditions. Hence, serum LPS concentration was evaluated in cirrhotic mice, and it was unveiled that both CCl_4_ and BDL models exhibited significantly elevated LPS levels versus the control group. Remarkably, rifaximin treatment effectively reduced serum LPS levels in both CCl_4_ and BDL mice (Fig 4A). To further reveal the effects of LPS on LSECs, primary LSECs were treated with LPS *in vitro*. Initially, a marked reduction was noteworthy in NO production by LSECs in the culture medium following LPS treatment (Fig 4B). Additionally, both eNOS and p-eNOS protein levels were significantly reduced in LSECs after exposure to LPS for 48h and across a range of concentrations (Figs 4C, D). However, there was subtle alteration in the p-eNOS/eNOS ratio (Fig 4C), suggesting that LPS have minor influence on the phosphorylation status of eNOS. Correspondingly, LPS treatment led to a downregulation of eNOS mRNA expression level (Fig 4E). These findings collectively indicated that LPS could inhibit NO production in LSECs primarily by modulating the transcriptional regulation of eNOS mRNA.

### LPS-induced LSEC dysfunction relied on TLR4 signaling

**Fig 5:**
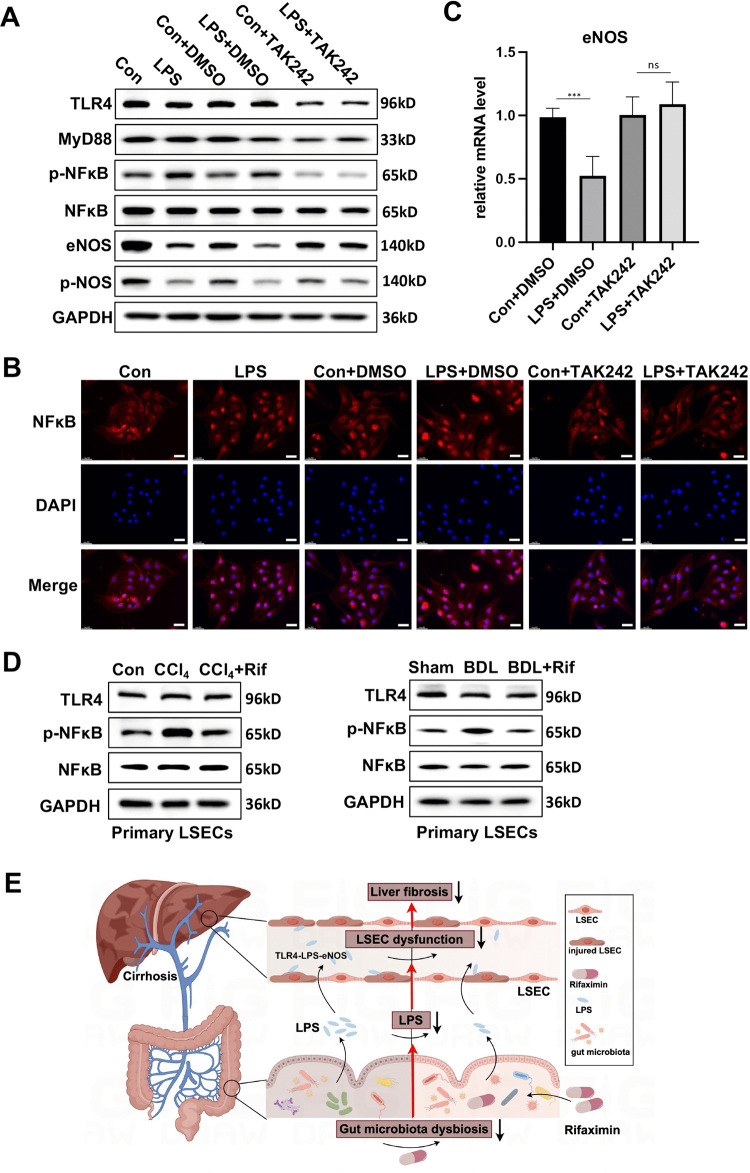
LPS-induced LSEC dysfunction relied on TLR4 signaling. **A.** Western blotting images of eNOS, p-eNOS, TLR4, MyD88, p-NF-κB, and NF-κB in primary LSECs incubated with or without TAK-242 for 2 h, followed by LPS treatment for 48 **h. B.** Immunocytochemistry staining of NF-κB in primary LSECs incubated with or without TAK-242 for 2 h, followed by LPS treatment for 48h. Scale bar:30 μm. **C.** The mRNA expression of eNOS in primary LSECs incubated with or without TAK-242 for 2h, followed by LPS treatment for 48h. All data are presented as mean ± SD, *P < 0.05, **P < 0.01, ***P < 0.001, ****P < 0.0001 (one-way ANOVA with Tukey’s post-hoc test). **D.** Western blotting images of TLR4, p-NF-κB, and NF-κB in primary LSECs isolated from CCl_4_ and BDL mice. GAPDH was used as a reference protein. **E.** The graphical abstract of this study. The intestinal microbiota dysbiosis allows more LPS to enter the portal circulation, which exacerbates LSEC dysfunction and liver fibrosis. LPS aggravates LSEC dysfunction by reducing eNOS expression which relies on TLR4 signaling. Intestinal decontamination with rifaximin improves LSEC function and alleviates liver fibrosis, a process linked to the reconstruction of the gut microbiome and a reduction in gut-derived LPS. LSECs, liver sinusoidal endothelial cells; LPS, lipopolysaccharide.

Given that TLR4 is the primary pattern recognition receptor for LPS, the next step was to indicate whether LPS-induced LSEC dysfunction could be mediated through the TLR4/MyD88/NFκB signaling pathway. Initially, LPS treatment resulted in increased phosphorylation of NFκB (p65) in primary LSECs (Fig 5A). Consistently, LPS treatment promoted the nuclear translocation of NFκB (p65) in primary LSECs, as demonstrated by cell immunofluorescence (Fig 5B). TAK242, a selective inhibitor of TLR4, was utilized to further probe this pathway. Treatment of LSECs with TAK242 significantly reduced the expression levels of TLR4, MyD88, and phosphorylated NFκB (p-p65), confirming the successful inhibition of TLR4 signaling (Figs 5A, B). Notably, the reduction in eNOS protein and mRNA levels induced by LPS was reversed following TLR4 signaling inhibition by TAK242 treatment (Figs 5A, C), indicating that LPS-induced LSEC dysfunction is TLR4-dependent. Consistently, rifaximin treatment decreased phosphorylated NFκB (p-p65) levels in LSECs in cirrhotic mice (Fig 5D).

## Discussion

The eNOS-derived NO, mainly produced by LSECs, is a major regulator of homeostasis in the liver, protecting HSCs from activation, dilating vascular tone of the hepatic microcirculatory system and inhibiting portal vein thrombosis [15,19]. LSEC dysfunction, mainly characterized by decreased production of eNOS-derived NO, could promote liver fibrosis and portal hypertension by activating HSCs and increasing vascular resistance [15]. The investigation into the regulator of LSEC dysfunction may provide potential targets for treating and preventing liver fibrosis. Gut microbiota and their metabolites represent potential therapeutic targets, given their entry into the liver sinusoid via portal circulation and their intimate interaction with LSECs. This study firstly demonstrated that intestinal decontamination with rifaximin could mitigate LSEC dysfunction in cirrhotic mice. While previous studies have shown rifaximin’s anti-fibrotic effects [36–41] and others have demonstrated the importance of LSECs in liver fibrosis [17], this work uniquely connects these two areas through a mechanistic pathway involving LPS-TLR4-eNOS axis, advancing our mechanistic understanding of the gut-liver axis by providing a pathway linking gut dysbiosis to LSEC dysfunction.

LPS, a pivotal bacterial-derived endotoxin, may translocate into the portal circulation due to increased intestinal permeability in cirrhotic conditions. Previous studies have indicated that serum LPS concentration was elevated in patients with CLD, correlating with gut microbial dysbiosis and compromised intestinal barrier integrity [42–44]. And rifaximin was reported to improve intestinal barrier function and decrease gut-derived LPS [28,39,45–47]. In alignment with these findings, the present study observed a significant elevation in serum LPS levels in cirrhotic mice, which was markedly attenuated by rifaximin treatment. Mechanistically, LPS exposure led to a pronounced downregulation of eNOS expression and a concomitant reduction in NO production by LSECs, implicating LPS as a key mediator of LSEC dysfunction. Intriguingly, LPS suppressed eNOS mRNA expression without directly impairing eNOS enzymatic activity, suggesting that LPS-induced LSEC dysfunction predominantly operates through transcriptional modulation of eNOS rather than post-translational modifications. However, the precise transcriptional regulators governing eNOS expression in response to LPS stimulation remain elusive and warrant further investigation. Given that NFκB activation is a key consequence of TLR4 signaling, it is a prime candidate. Future studies employing techniques such as chromatin immunoprecipitation (ChIP) assays are needed to determine if NFκB directly binds to the eNOS promoter or acts through intermediary factors.

Toll-like receptor 4 (TLR4) is a pivotal component of the immune system, serving as a key pattern recognition receptor (PRR) that detects pathogen-associated molecular patterns (PAMPs) [48]. LPS is the most well-known ligand for TLR4. Upon activation by LPS, TLR4 recruits MyD88 and then activates NFκB signaling pathway, initiating a cascade of pro-inflammatory factors [48]. TLR4 is mainly expressed in immune cells, while previous studies have confirmed that TLR4 is highly expressed in LSECs [49, 50]. Jagavelu et al. demonstrated that TLR4-mutant mice (C3H/HeJ, TLR4-MT) exhibited impaired angiogenesis in liver endothelial cells and a concomitant reduction in liver fibrosis, highlighting the pivotal role of TLR4 signaling in hepatic pathophysiology [50]. In the present study, utilizing primary murine LSECs, TLR4/MyD88/NFκB signaling pathway was identified as a critical pathway mediating LPS-induced LSEC dysfunction *in vitro*. Given that the liver serves as the principal site for LPS clearance, with LSECs accounting for approximately 75% of this process [51], emerging evidence has redefined the mechanistic framework by which LPS is processed. A recent study highlighted that Stab1, rather than TLR4, serves as the predominant receptor facilitating LPS endocytosis and clearance in LSECs[49], whereas TLR4 primarily functions as a receptor that detects LPS and triggers inflammatory signaling cascades. Notably, these receptors can exert functionally antagonistic roles in LPS homeostasis, and Stab1-mediated clearance may serve as a critical defense mechanism to mitigate excessive TLR4 activation and subsequent inflammatory responses [49]. In alignment with these findings, the present study revealed that LPS stimulation remarkably activated NFκB via TLR4 in LSECs, highlighting the pro-inflammatory role of TLR4 signaling. Moreover, Stab1 expression level was downregulated in LSECs in fibrotic liver [25], suggesting a pathological shift in LPS handling that may exacerbate hepatic inflammation. These findings support a mechanistic model, in which gut microbial dysbiosis drives excessive LPS influx into the liver, overwhelming the clearance capacity of Stab1 and enhancing TLR4 signaling in LSECs. This elevated TLR4 activation, in turn, contributes to LSEC dysfunction by suppressing eNOS expression, potentially providing a microenvironment conducive to liver fibrosis progression and the exacerbation of portal hypertension. However, definitive elucidation of the role of TLR4 signaling in LSECs in the gut-liver axis and its contribution to hepatic fibrogenesis necessitates further investigation using LSEC-specific TLR4 knockout models.

Rifaximin is a broad-spectrum, gut-selective antibiotic with minimal systemic absorption, making it as an optimal candidate for modulating intestinal microbiota while minimizing extraneous systemic effects [52]. In the present study, rifaximin was preferentially employed over other broad-spectrum antibiotics based on two principal considerations. Firstly, its non-systemic pharmacokinetics allow for targeted modulation of gut microbiota, thereby enabling a precise evaluation of gut-liver interactions without the confounding influence of systemic antibiotic exposure. Secondly, rifaximin has been extensively utilized in the clinical management of hepatic encephalopathy (HE), a prevalent complication of liver cirrhosis [37,53], and accumulating evidence suggests its potential to mitigate portal hypertension in cirrhotic patients [54,55]. The anti-fibrotic effect of rifaximin have been reported in previous studies [40,41], which align with the findings of the present study. These findings highlight the potential therapeutic value of rifaximin in liver cirrhosis beyond its established role in HE management, necessitating further clinical exploration to delineate its broader implications.

The gut microbiota composition in cirrhotic mice exhibited remarkable dysbiosis versus controls, a perturbation that was partially reversed following rifaximin administration. This microbiota modulation may underlie rifaximin’s beneficial effects on liver fibrosis and LSEC dysfunction. Notably, key probiotic taxa, including *Lactobacillus apodemi, Lactobacillus murinus*, and *Bifidobacterium pseudolongum*, which were significantly diminished in cirrhotic mice, were re-enriched post-treatment. The functional relevance of these bacterial species has been highlighted in previous studies, wherein extracellular vesicles derived from *Lactobacillus murinus* were found to ameliorate intestinal barrier dysfunction in murine models [56], while *Lactobacillus murinus* itself attenuated hepatic inflammation and fibrosis in primary sclerosing cholangitis (PSC) mice [57]. Similarly, *Bifidobacterium pseudolongum* has been implicated in intestinal barrier preservation [58] and hepatoprotection [59]. Conversely, rifaximin treatment led to a significant depletion of taxa, such as *Romboutsia, Frisingicoccus*, and *Turicibacter*, which were markedly enriched in cirrhotic mice. It would be of value to perform correlation analyses to determine whether these bacterial taxa are associated with key phenotypic or molecular parameters, such as serum LPS levels or eNOS expression in LSECs. Unfortunately, such an analysis was precluded in the current study because serum, liver tissue and fecal samples were not tracked and matched on an individual mouse during collection. Future studies employing coordinated longitudinal sampling from the same subjects will be essential to establish direct links between specific microbial changes and host pathophysiology within the gut-liver axis.

While this study provided evidence supporting the novel connection between gut microbiota and LSEC function, several limitations should be acknowledged. Firstly, the study was conducted entirely in murine models. While the CCl₄ and BDL models were well-established for studying liver fibrosis, they may not fully capture the complexity of human liver disease, particularly with respect to etiological diversity and host-microbiome interactions. Future studies should focus on validating these finding in human contexts. Secondly, although rifaximin’s low systemic absorption supported a gut-mediated mechanism, we cannot exclude potential indirect effects by rifaximin, such as alterations in bile acid metabolism, suppression of Kupffer cell activation, or amelioration of systemic inflammation, which may also contribute to the observed hepatic improvements. To establish a causal relationship between gut microbiota and LSEC dysfunction, future investigations could employ germ-free mice and fecal microbiota transplantation (FMT) approaches in addition to antibiotic interventions. Furthermore, while our study identified LPS as a key microbial mediator of LSEC dysfunction, we recognized that this represents only one component of a complex landscape of gut-derived products. Other specific translocated bacterial species or metabolites and their respective receptors may also directly contribute to LSEC dysfunction. The Multi-omics analysis including metabolomics analysis of blood, metagenomic sequencing of gut microbiota and transcriptomics analysis of LSECs would provide a more comprehensive perspective and help identify additional microbial contributors to LSEC injury and protection.

## Conclusions

Gut microbial dysbiosis and gut-derived LPS might aggravate LSEC dysfunction and liver fibrosis. Rifaximin treatment ameliorated LSEC dysfunction and liver fibrosis, which might be associated with the reconstruction of the gut microbiome and a reduction in gut-derived LPS in the liver (Fig 5E). This study revealed that gut microbial manipulation may be a promising strategy to alleviate LSEC dysfunction, emerging crucial for the prevention and treatment of liver cirrhosis.

## Supporting information

S1 Fig Sequencing depth and Rarefaction curves of 16S rRNA analysis. A. Table showing the sequencing depth of each sample in 16S rRNA analysis. **B.** Rarefaction curves of 16S rRNA analysis.
(PDF)

S1 Data Raw Images. (PDF)

## References

[1] Smith A, Baumgartner K, Bositis C. Cirrhosis: diagnosis and management. Am Fam Physician. 2019;100(12):759–70. 31845776

[2] Friedman SL. Mechanisms of hepatic fibrogenesis. Gastroenterology. 2008;134(6):1655–69. doi: 10.1053/j.gastro.2008.03.003 18471545 PMC2888539

[3] Trebicka J, Bork P, Krag A, Arumugam M. Utilizing the gut microbiome in decompensated cirrhosis and acute-on-chronic liver failure. Nat Rev Gastroenterol Hepatol. 2021;18(3):167–80. doi: 10.1038/s41575-020-00376-3 33257833

[4] Trebicka J, Macnaughtan J, Schnabl B, Shawcross DL, Bajaj JS. The microbiota in cirrhosis and its role in hepatic decompensation. J Hepatol. 2021;75 Suppl 1(Suppl 1):S67–81. doi: 10.1016/j.jhep.2020.11.013 34039493 PMC8973011

[5] Schnabl B, Brenner DA. Interactions between the intestinal microbiome and liver diseases. Gastroenterology. 2014;146(6):1513–24. doi: 10.1053/j.gastro.2014.01.020 24440671 PMC3996054

[6] Bajaj JS. Alcohol, liver disease and the gut microbiota. Nat Rev Gastroenterol Hepatol. 2019;16(4):235–46. doi: 10.1038/s41575-018-0099-1 30643227

[7] Bajaj JS, Heuman DM, Hylemon PB, Sanyal AJ, White MB, Monteith P, et al. Altered profile of human gut microbiome is associated with cirrhosis and its complications. J Hepatol. 2014;60(5):940–7. doi: 10.1016/j.jhep.2013.12.019 24374295 PMC3995845

[8] Qin N, Yang F, Li A, Prifti E, Chen Y, Shao L, et al. Alterations of the human gut microbiome in liver cirrhosis. Nature. 2014;513(7516):59–64. doi: 10.1038/nature13568 25079328

[9] Oh TG, Kim SM, Caussy C, Fu T, Guo J, Bassirian S, et al. A Universal Gut-Microbiome-Derived Signature Predicts Cirrhosis. Cell Metab. 2020;32(5):878-888.e6. doi: 10.1016/j.cmet.2020.06.005 32610095 PMC7822714

[10] Chen P, Stärkel P, Turner JR, Ho SB, Schnabl B. Dysbiosis-induced intestinal inflammation activates tumor necrosis factor receptor I and mediates alcoholic liver disease in mice. Hepatology. 2015;61(3):883–94. doi: 10.1002/hep.27489 25251280 PMC4340725

[11] Schwabl P, Hambruch E, Seeland BA, Hayden H, Wagner M, Garnys L, et al. The FXR agonist PX20606 ameliorates portal hypertension by targeting vascular remodelling and sinusoidal dysfunction. J Hepatol. 2017;66(4):724–33. doi: 10.1016/j.jhep.2016.12.005 27993716

[12] Schirbel A, Kessler S, Rieder F, West G, Rebert N, Asosingh K, et al. Pro-angiogenic activity of TLRs and NLRs: a novel link between gut microbiota and intestinal angiogenesis. Gastroenterology. 2013;144(3):613-623.e9. doi: 10.1053/j.gastro.2012.11.005 23149220 PMC3578104

[13] Chu H, Duan Y, Lang S, Jiang L, Wang Y, Llorente C, et al. The Candida albicans exotoxin candidalysin promotes alcohol-associated liver disease. J Hepatol. 2020;72(3):391–400. doi: 10.1016/j.jhep.2019.09.029 31606552 PMC7031049

[14] Pradere J-P, Troeger JS, Dapito DH, Mencin AA, Schwabe RF. Toll-like receptor 4 and hepatic fibrogenesis. Semin Liver Dis. 2010;30(3):232–44. doi: 10.1055/s-0030-1255353 20665376 PMC4099360

[15] McConnell MJ, Kostallari E, Ibrahim SH, Iwakiri Y. The evolving role of liver sinusoidal endothelial cells in liver health and disease. Hepatology. 2023;78(2):649–69. doi: 10.1097/HEP.0000000000000207 36626620 PMC10315420

[16] Xie G, Wang X, Wang L, Wang L, Atkinson RD, Kanel GC, et al. Role of differentiation of liver sinusoidal endothelial cells in progression and regression of hepatic fibrosis in rats. Gastroenterology. 2012;142(4):918-927.e6. doi: 10.1053/j.gastro.2011.12.017 22178212 PMC3618963

[17] Poisson J, Lemoinne S, Boulanger C, Durand F, Moreau R, Valla D, et al. Liver sinusoidal endothelial cells: Physiology and role in liver diseases. J Hepatol. 2017;66(1):212–27. doi: 10.1016/j.jhep.2016.07.009 27423426

[18] Shah V, Haddad FG, Garcia-Cardena G, Frangos JA, Mennone A, Groszmann RJ, et al. Liver sinusoidal endothelial cells are responsible for nitric oxide modulation of resistance in the hepatic sinusoids. J Clin Invest. 1997;100(11):2923–30. doi: 10.1172/JCI119842 9389760 PMC508500

[19] Gao J, Lan T, Kostallari E, Guo Y, Lai E, Guillot A, et al. Angiocrine signaling in sinusoidal homeostasis and liver diseases. J Hepatol. 2024;81(3):543–61. doi: 10.1016/j.jhep.2024.05.014 38763358 PMC11906189

[20] Kiouptsi K, Pontarollo G, Reinhardt C. Gut Microbiota and the Microvasculature. Cold Spring Harb Perspect Med. 2023;13(8):a041179. doi: 10.1101/cshperspect.a041179 37460157 PMC10411863

[21] Wang Y, Zhang Y, Liu Y, Xu J, Liu Y. Gut-liver axis: liver sinusoidal endothelial cells function as the hepatic barrier in colitis-induced liver injury. Front Cell Dev Biol. 2021;9:702890. doi: 10.3389/fcell.2021.702890 34336855 PMC8322652

[22] Formes H, Bernardes JP, Mann A, Bayer F, Pontarollo G, Kiouptsi K, et al. The gut microbiota instructs the hepatic endothelial cell transcriptome. iScience. 2021;24(10):103092. doi: 10.1016/j.isci.2021.103092 34622147 PMC8479694

[23] Cogger VC, Mohamad M, Solon-Biet SM, Senior AM, Warren A, O’Reilly JN, et al. Dietary macronutrients and the aging liver sinusoidal endothelial cell. Am J Physiol Heart Circ Physiol. 2016;310(9):H1064-70. doi: 10.1152/ajpheart.00949.2015 26921440

[24] Cheluvappa R, Jamieson HA, Hilmer SN, Muller M, Le Couteur DG. The effect of Pseudomonas aeruginosa virulence factor, pyocyanin, on the liver sinusoidal endothelial cell. J Gastroenterol Hepatol. 2007;22(8):1350–1. 17688676 10.1111/j.1440-1746.2007.05016.x

[25] Su T, Yang Y, Lai S, Jeong J, Jung Y, McConnell M, et al. Single-cell transcriptomics reveals zone-specific alterations of liver sinusoidal endothelial cells in cirrhosis. Cell Mol Gastroenterol Hepatol. 2021;11(4):1139–61. doi: 10.1016/j.jcmgh.2020.12.007 33340713 PMC7903131

[26] Ikeuchi K, Tsutsumi T, Ishizaka A, Mizutani T, Sedohara A, Koga M, et al. Modulation of duodenal and jejunal microbiota by rifaximin in mice with CCl4-induced liver fibrosis. Gut Pathog. 2023;15(1):14. doi: 10.1186/s13099-023-00541-4 36945059 PMC10029291

[27] Yang L, Liu B, Zheng J, Huang J, Zhao Q, Liu J. Rifaximin alters intestinal microbiota and prevents progression of ankylosing spondylitis in mice. Front Cell Infect Microbiol. 2019;9:44. 30886835 10.3389/fcimb.2019.00044 PMC6409347

[28] Du L, Jiang W, Zhu X, Zhu L, Fan Y, Jiang W. Rifaximin alleviates intestinal barrier disruption and systemic inflammation via the PXR/NFκB/MLCK pathway and modulates intestinal Lachnospiraceae abundance in heat-stroke mice. Int Immunopharmacol. 2024;143(Pt 2):113462. doi: 10.1016/j.intimp.2024.113462 39461239

[29] Cabral F, Miller CM, Kudrna KM, Hass BE, Daubendiek JG, Kellar BM, et al. Purification of Hepatocytes and Sinusoidal Endothelial Cells from Mouse Liver Perfusion. J Vis Exp. 2018(132). 10.3791/56993 PMC5894826 29553556

[30] Liu C, Zhao D, Ma W, Guo Y, Wang A, Wang Q, et al. Denitrifying sulfide removal process on high-salinity wastewaters in the presence of Halomonas sp. Appl Microbiol Biotechnol. 2016;100(3):1421–6. doi: 10.1007/s00253-015-7039-6 26454867

[31] Chen S, Zhou Y, Chen Y, Gu J. fastp: an ultra-fast all-in-one FASTQ preprocessor. Bioinformatics. 2018;34(17):i884–90. doi: 10.1093/bioinformatics/bty560 30423086 PMC6129281

[32] Magoč T, Salzberg SL. FLASH: fast length adjustment of short reads to improve genome assemblies. Bioinformatics. 2011;27(21):2957–63. doi: 10.1093/bioinformatics/btr507 21903629 PMC3198573

[33] Edgar RC. UPARSE: highly accurate OTU sequences from microbial amplicon reads. Nat Methods. 2013;10(10):996–8. doi: 10.1038/nmeth.2604 23955772

[34] Wang Q, Garrity GM, Tiedje JM, Cole JR. Naive Bayesian classifier for rapid assignment of rRNA sequences into the new bacterial taxonomy. Appl Environ Microbiol. 2007;73(16):5261–7. doi: 10.1128/AEM.00062-07 17586664 PMC1950982

[35] Schloss PD, Westcott SL, Ryabin T, Hall JR, Hartmann M, Hollister EB, et al. Introducing mothur: open-source, platform-independent, community-supported software for describing and comparing microbial communities. Appl Environ Microbiol. 2009;75(23):7537–41. doi: 10.1128/AEM.01541-09 19801464 PMC2786419

[36] Israelsen M, Madsen BS, Torp N, Johansen S, Hansen CD, Detlefsen S, et al. Rifaximin-α for liver fibrosis in patients with alcohol-related liver disease (GALA-RIF): a randomised, double-blind, placebo-controlled, phase 2 trial. Lancet Gastroenterol Hepatol. 2023;8(6):523–32. doi: 10.1016/S2468-1253(23)00010-9 36893774 PMC10172147

[37] Caraceni P, Vargas V, Solà E, Alessandria C, de Wit K, Trebicka J, et al. The use of rifaximin in patients with cirrhosis. Hepatology. 2021;74(3):1660–73. doi: 10.1002/hep.31708 33421158 PMC8518409

[38] Israelsen M, Torp N, Johansen S, Thiele M, Krag A, GALAXY and MicrobLiver consortia. Rifaximin-α for liver fibrosis in patients with alcohol-related liver disease - Authors’ reply. Lancet Gastroenterol Hepatol. 2023;8(7):604. doi: 10.1016/S2468-1253(23)00155-3 37301207

[39] Enomoto M, Kaji K, Nishimura N, Fujimoto Y, Murata K, Takeda S, et al. Rifaximin and lubiprostone mitigate liver fibrosis development by repairing gut barrier function in diet-induced rat steatohepatitis. Dig Liver Dis. 2022;54(10):1392–402. doi: 10.1016/j.dld.2022.04.012 35514019

[40] Oguri N, Miyoshi J, Nishinarita Y, Wada H, Nemoto N, Hibi N, et al. Akkermansia muciniphila in the small intestine improves liver fibrosis in a murine liver cirrhosis model. NPJ Biofilms Microbiomes. 2024;10(1):81. doi: 10.1038/s41522-024-00564-y 39285193 PMC11405509

[41] Zhu Q, Zou L, Jagavelu K, Simonetto DA, Huebert RC, Jiang Z-D, et al. Intestinal decontamination inhibits TLR4 dependent fibronectin-mediated cross-talk between stellate cells and endothelial cells in liver fibrosis in mice. J Hepatol. 2012;56(4):893–9. doi: 10.1016/j.jhep.2011.11.013 22173161 PMC3307873

[42] Harte AL, da Silva NF, Creely SJ, McGee KC, Billyard T, Youssef-Elabd EM, et al. Elevated endotoxin levels in non-alcoholic fatty liver disease. J Inflamm (Lond). 2010;7:15. doi: 10.1186/1476-9255-7-15 20353583 PMC2873499

[43] Michelena J, Altamirano J, Abraldes JG, Affò S, Morales-Ibanez O, Sancho-Bru P, et al. Systemic inflammatory response and serum lipopolysaccharide levels predict multiple organ failure and death in alcoholic hepatitis. Hepatology. 2015;62(3):762–72. doi: 10.1002/hep.27779 25761863 PMC4549175

[44] Bigatello LM, Broitman SA, Fattori L, Di Paoli M, Pontello M, Bevilacqua G, et al. Endotoxemia, encephalopathy, and mortality in cirrhotic patients. Am J Gastroenterol. 1987;82(1):11–5. 3799574

[45] Zeng X, Tang XJ, Sheng X, Ni W, Xin HG, Chen WZ, et al. Does low-dose rifaximin ameliorate endotoxemia in patients with liver cirrhosis: a prospective study. J Dig Dis. 2015;16(11):665–74. doi: 10.1111/1751-2980.12294 26474237

[46] Bajaj JS, Heuman DM, Sanyal AJ, Hylemon PB, Sterling RK, Stravitz RT, et al. Modulation of the metabiome by rifaximin in patients with cirrhosis and minimal hepatic encephalopathy. PLoS One. 2013;8(4):e60042. doi: 10.1371/journal.pone.0060042 23565181 PMC3615021

[47] Qiu T, Zhu X, Wu J, Hong W, Hu W, Fang T. Mechanisms of rifaximin inhibition of hepatic fibrosis in mice with metabolic dysfunction associated steatohepatitis through the TLR4/NFκB pathway. Sci Rep. 2025;15(1):9815. doi: 10.1038/s41598-025-92282-4 40118973 PMC11928543

[48] Kim H-J, Kim H, Lee J-H, Hwangbo C. Toll-like receptor 4 (TLR4): new insight immune and aging. Immun Ageing. 2023;20(1):67. doi: 10.1186/s12979-023-00383-3 38001481 PMC10668412

[49] Cabral F, Al-Rahem M, Skaggs J, Thomas TA, Kumar N, Wu Q, et al. Stabilin receptors clear LPS and control systemic inflammation. iScience. 2021;24(11):103337. doi: 10.1016/j.isci.2021.103337 34816100 PMC8591421

[50] Jagavelu K, Routray C, Shergill U, O’Hara SP, Faubion W, Shah VH. Endothelial cell toll-like receptor 4 regulates fibrosis-associated angiogenesis in the liver. Hepatology. 2010;52(2):590–601. doi: 10.1002/hep.23739 20564354 PMC2916032

[51] DeLeve LD, Maretti-Mira AC. Liver sinusoidal endothelial cell: an update. Seminars in Liver Disease. 2017;37(4):377–87. 29272898 10.1055/s-0037-1617455 PMC6005648

[52] Scarpignato C, Pelosini I. Rifaximin, a poorly absorbed antibiotic: pharmacology and clinical potential. Chemotherapy. 2005;51 Suppl 1:36–66. doi: 10.1159/000081990 15855748

[53] Bass NM, Mullen KD, Sanyal A, Poordad F, Neff G, Leevy CB, et al. Rifaximin treatment in hepatic encephalopathy. N Engl J Med. 2010;362(12):1071–81. doi: 10.1056/NEJMoa0907893 20335583

[54] Vlachogiannakos J, Saveriadis AS, Viazis N, Theodoropoulos I, Foudoulis K, Manolakopoulos S, et al. Intestinal decontamination improves liver haemodynamics in patients with alcohol-related decompensated cirrhosis. Aliment Pharmacol Ther. 2009;29(9):992–9. doi: 10.1111/j.1365-2036.2009.03958.x 19210289

[55] Lim YL, Kim MY, Jang YO, Baik SK, Kwon SO. Rifaximin and propranolol combination therapy is more effective than propranolol monotherapy for the reduction of portal pressure: an open randomized controlled pilot study. Gut Liver. 2017;11(5):702–10. doi: 10.5009/gnl16478 28651304 PMC5593333

[56] Fan J, Zhang Y, Zuo M, Ding S, Li J, Feng S, et al. Novel mechanism by which extracellular vesicles derived from Lactobacillus murinus alleviates deoxynivalenol-induced intestinal barrier disruption. Environ Int. 2024;185:108525. doi: 10.1016/j.envint.2024.108525 38408410

[57] Shen Y, Jiang B, Zhang C, Wu Q, Li L, Jiang P. Combined inhibition of the TGF-beta1/Smad pathway by Prevotella copri and Lactobacillus murinus to reduce inflammation and fibrosis in primary sclerosing cholangitis. Int J Mol Sci. 2023;24(13). 10.3390/ijms241311010 PMC10341692 37446187

[58] Guo W, Mao B, Cui S, Tang X, Zhang Q, Zhao J. Protective effects of a novel probiotic Bifidobacterium pseudolongum on the intestinal barrier of colitis mice via modulating the Ppargamma/STAT3 pathway and intestinal microbiota. Foods. 2022;11(11). 10.3390/foods11111551 PMC9180506 35681301

[59] Guo W, Cui S, Tang X, Yan Y, Xiong F, Zhang Q, et al. Intestinal microbiomics and hepatic metabolomics insights into the potential mechanisms of probiotic Bifidobacterium pseudolongum CCFM1253 preventing acute liver injury in mice. J Sci Food Agric. 2023;103(12):5958–69. doi: 10.1002/jsfa.12665 37099000

