# Supplementary material for: Intestinal decontamination with rifaximin attenuates LSEC dysfunction and liver fibrosis in mice
Source: PLoS One. 2026 Jan 23;21(1):e0340664. doi: 10.1371/journal.pone.0340664 (PMC12829844; doi:10.1371/journal.pone.0340664)

A

SampleInfo	Seq_num	Base_num	Mean_length	Min_length	Max_length
CCl4_1	62286	26095406	418.96102	258	432
CCl4_2	62092	26082322	420.0593	262	505
CCl4_3	53564	22379745	417.81318	235	430
CCl4_4	61469	25541280	415.51481	317	431
CCl4_5	62172	25872830	416.14923	269	432
CCl4_Rif1	52667	22087145	419.37352	317	506
CCl4_Rif2	67474	28370599	420.46713	309	517
CCl4_Rif3	71225	30001888	421.22693	251	445
CCl4_Rif4	75398	31726836	420.79148	300	437
CCl4_Rif5	67125	28137729	419.18405	234	441
con1	55476	23350258	420.90738	318	431
con2	115152	48326623	419.6768	203	445
con3	61956	26076647	420.88978	316	434
con4	71961	29924066	415.83727	261	440
con5	70480	29430343	417.57013	282	486
×Ü¼Æ(15)	1010497	423403717	418.96146733333	203	517

B

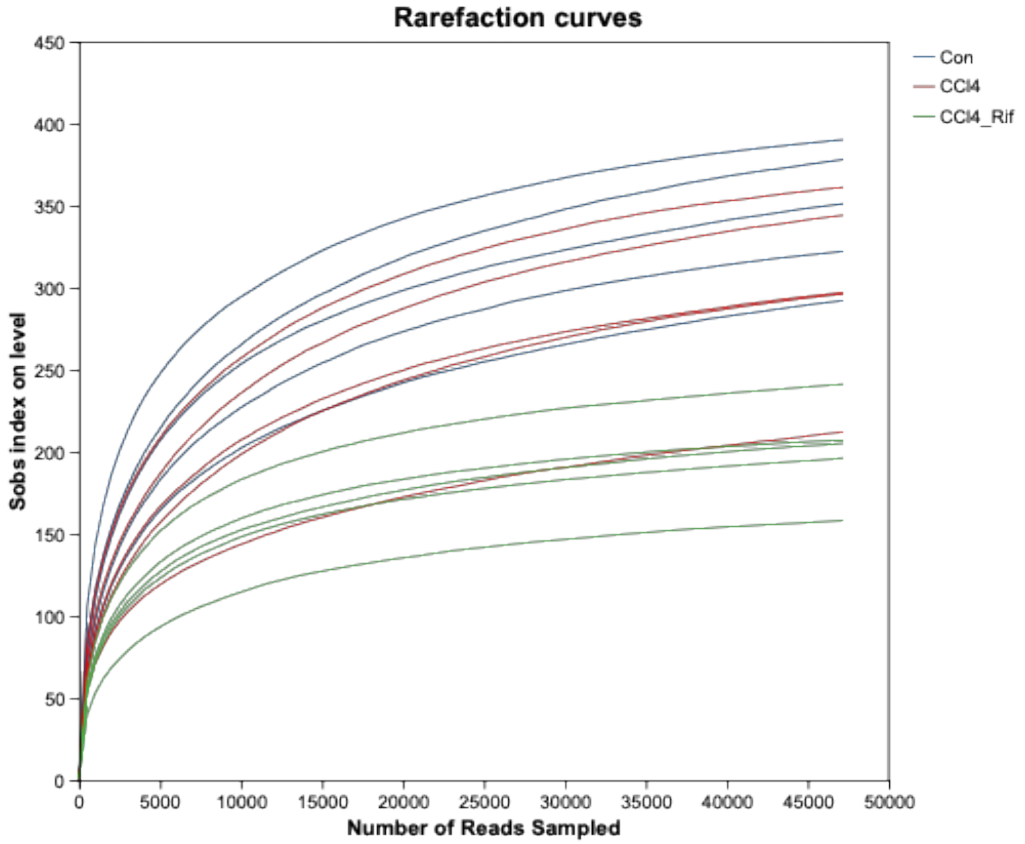

Supplement: S1 Fig — A. Table showing the sequencing depth of each sample in 16S rRNA analysis. B. Rarefaction curves of 16S rRNA analysis. (PDF) [file pone.0340664.s001.pdf]
