# Supplementary material for: Intestinal decontamination with rifaximin attenuates LSEC dysfunction and liver fibrosis in mice
Source: PLoS One. 2026 Jan 23;21(1):e0340664. doi: 10.1371/journal.pone.0340664 (PMC12829844; doi:10.1371/journal.pone.0340664)

Fig 1E ECL

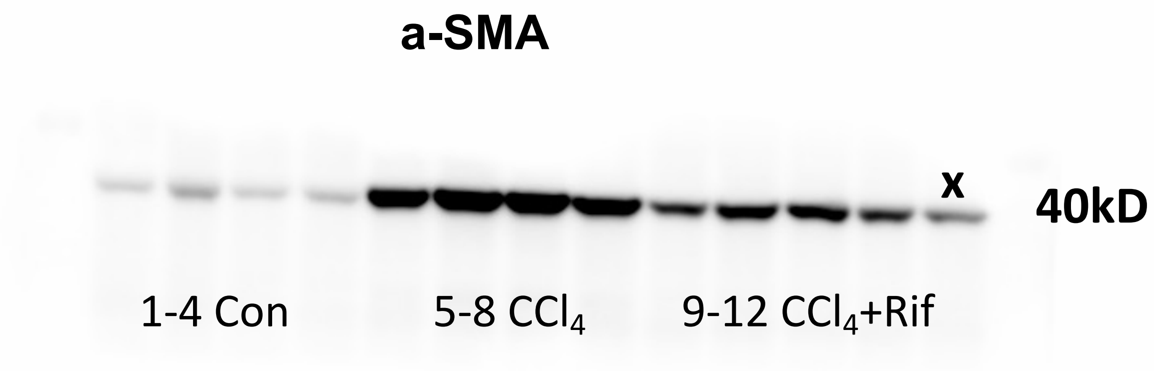

Fig 1E   ECL

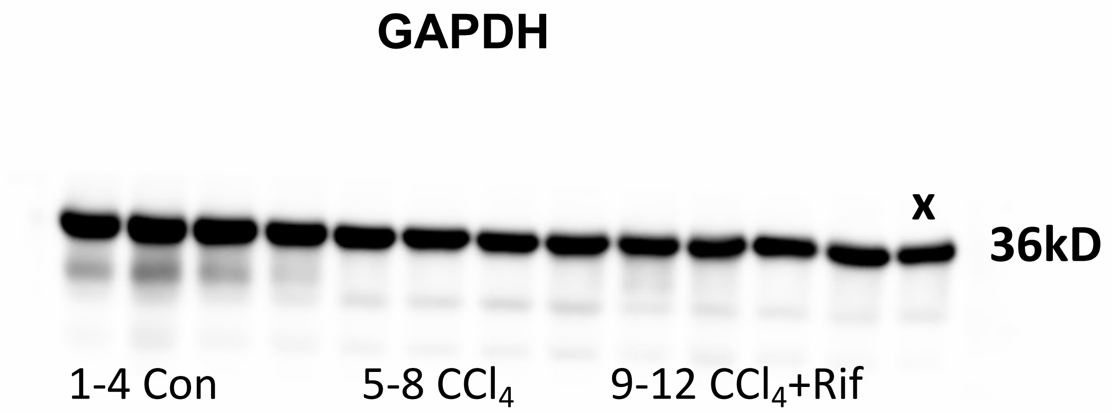

Fig 1E ECL

a-SMA

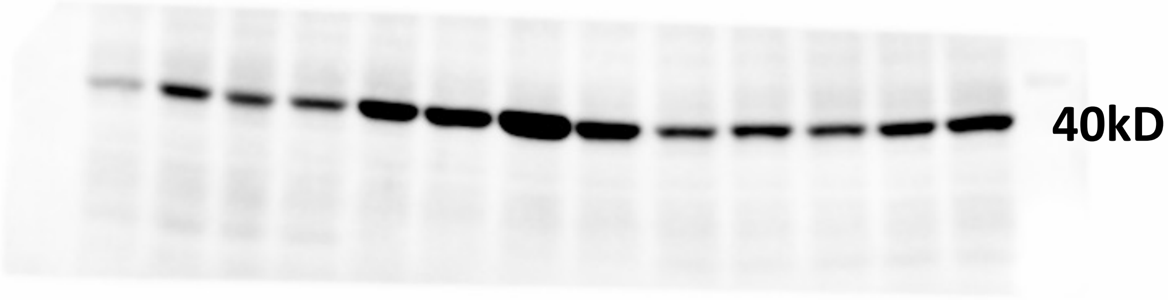

1-4 Con 5-8 BDL 9-13BDL+Rif

Fig 1E ECL

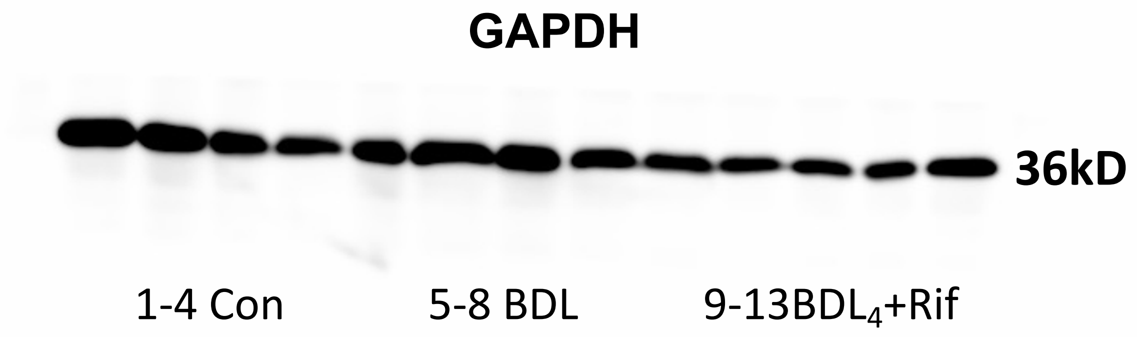

Fig 2C ECL

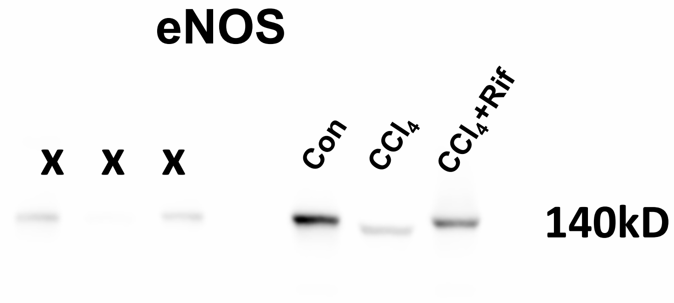

Fig 2C ECL

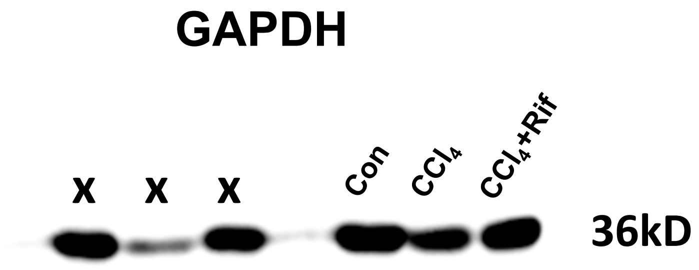

Fig 2C ECL

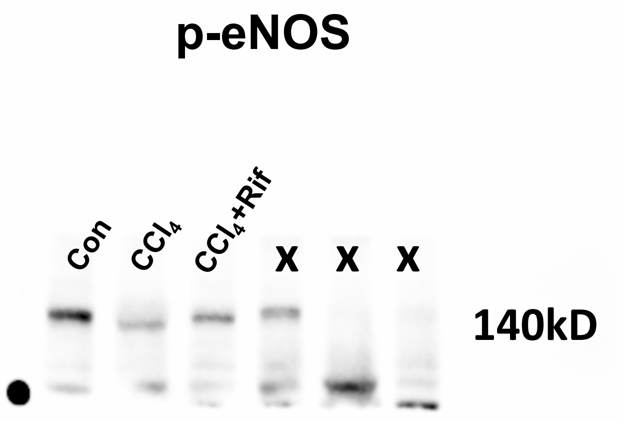

Fig 2D ECL

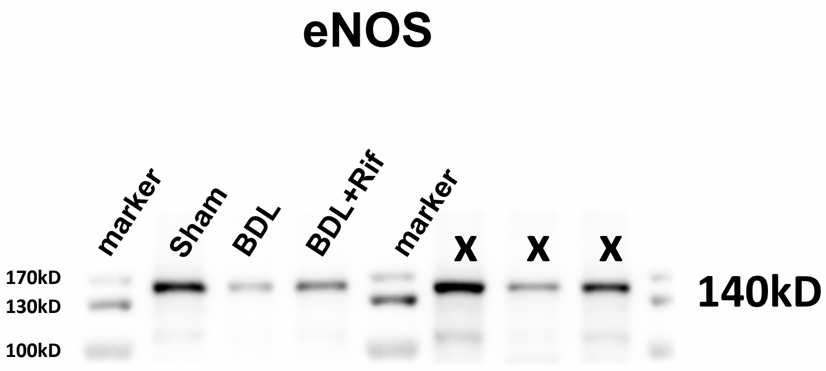

eNOS
marker
Sham
BDL
BDL+Rif
marker
X
X
X
170kD
130kD
100kD
140kD

Fig 2D ECL

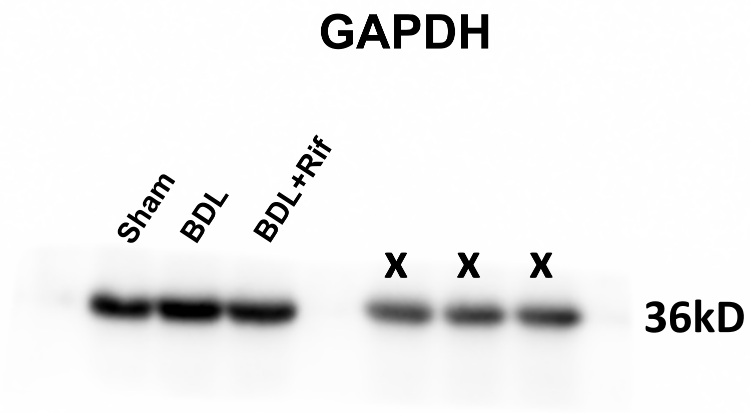

Fig 2D ECL

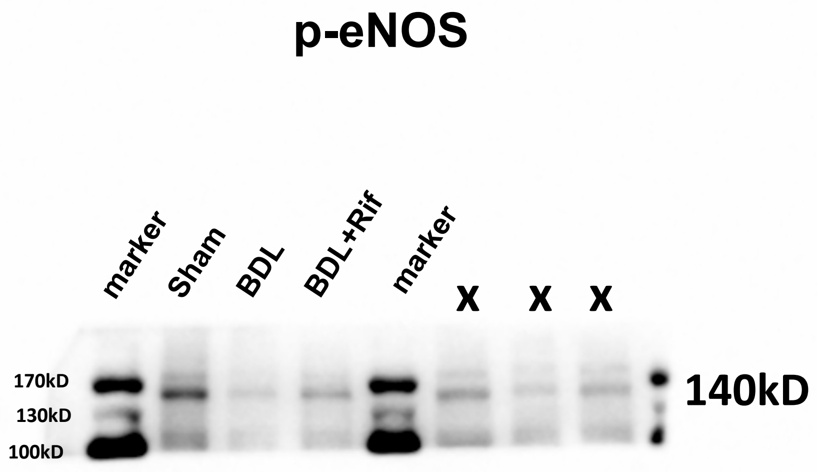

p-eNOS
marker
Sham
BDL
BDL+Rif
marker
x
x
x
170kD
130kD
100kD
140kD

Fig 4C ECL

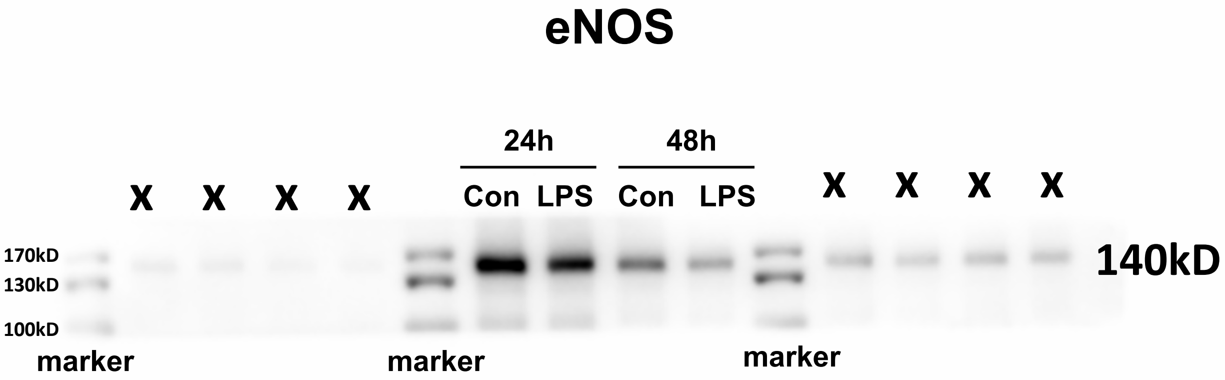
eNOS
24h
48h
x
x
x
x
Con
LPS
Con
LPS
x
x
x
x
170kD
130kD
100kD
140kD
marker
marker
marker

Fig 4C ECL

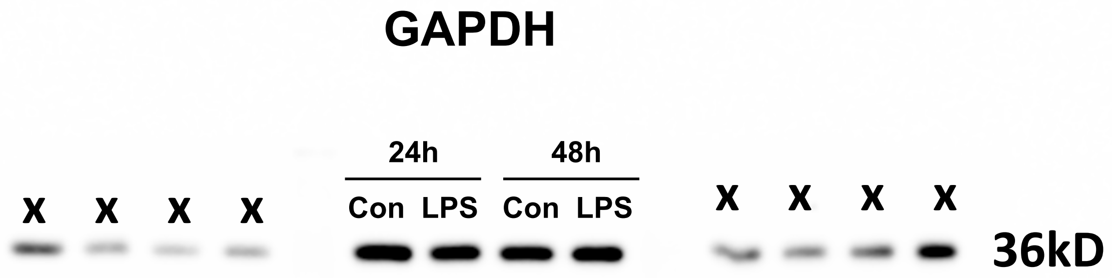

Fig 4C ECL

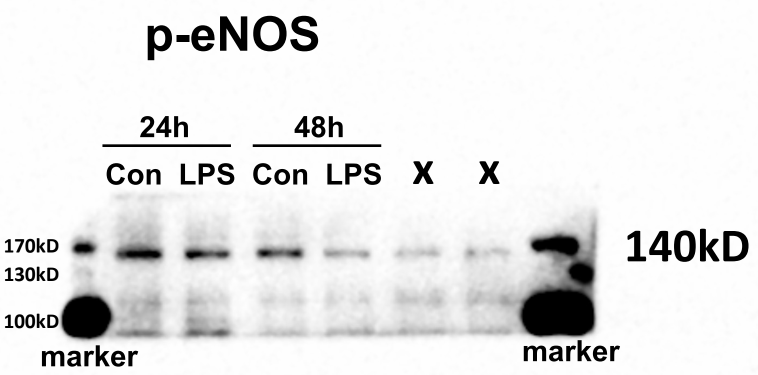
p-eNOS
24h
48h
Con LPS Con LPS x x
170kD
130kD
100kD
140kD
marker
marker

Fig 4D ECL

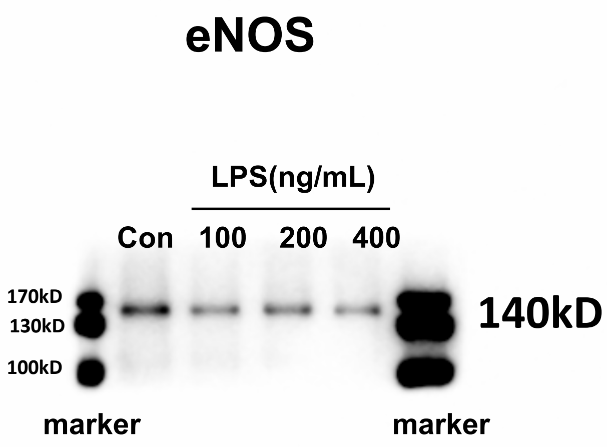
eNOS
LPS(ng/mL)
Con
100
200
400
170kD
130kD
100kD
140kD
marker
marker

Fig 4D ECL

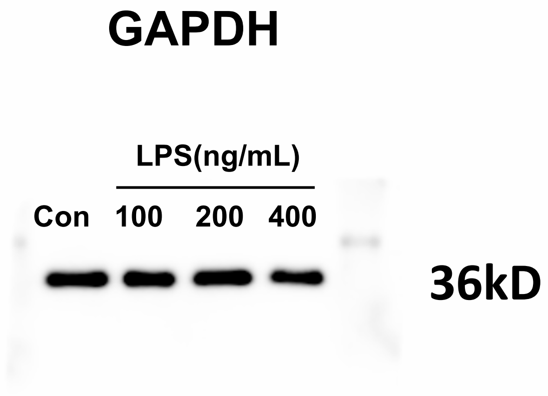

Fig 4D ECL

p-eNOS

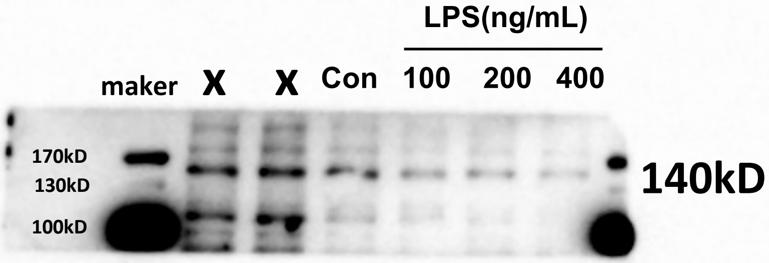

Fig 5A ECL

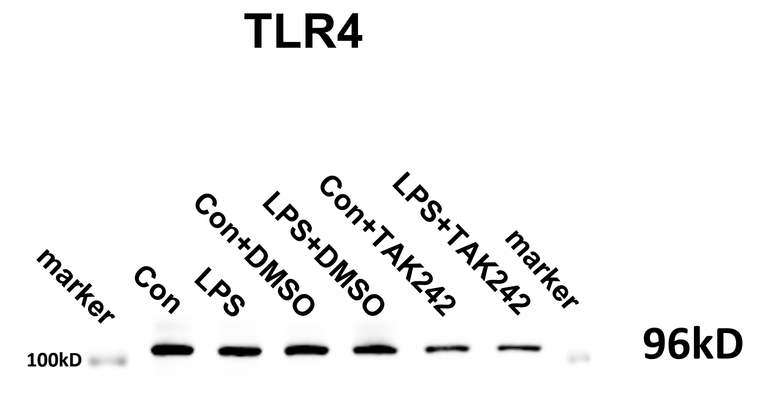
TLR4
marker
Con
LPS
Con+DMSO
LPS+DMSO
Con+TAK242
LPS+TAK242
marker
100kD
96kD

Fig 5A ECL

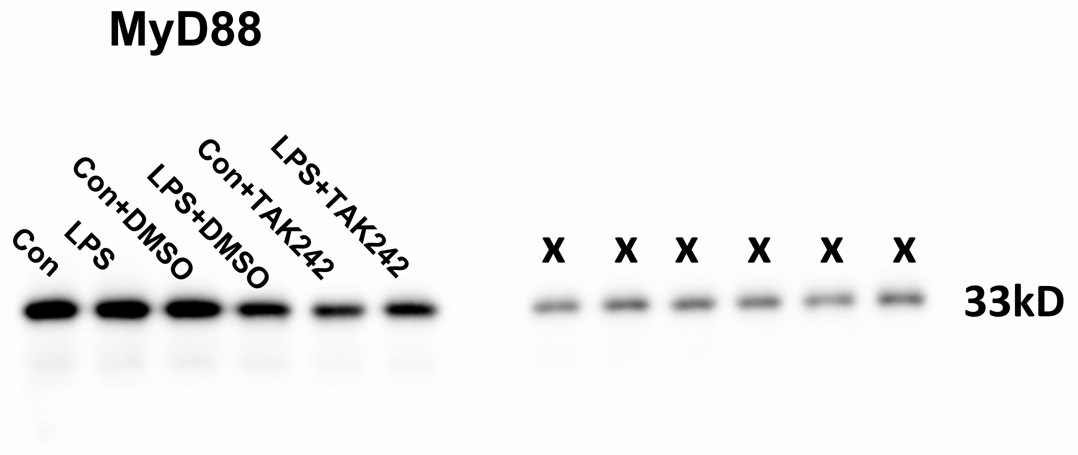
MyD88
Con
LPS
Con+DMSO
LPS+DMSO
Con+TAK242
LPS+TAK242
x
x
x
x
x
x
33kD

Fig 5A ECL

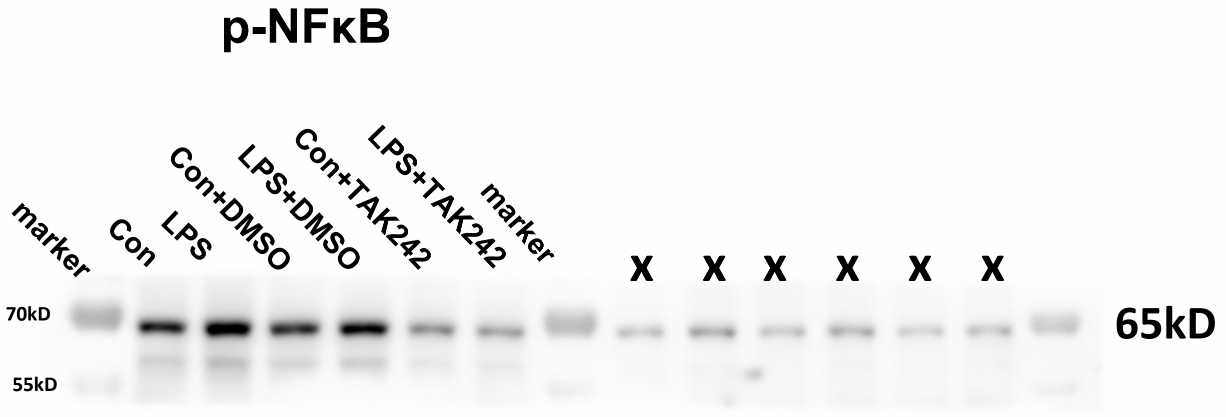

p-NFκB
marker
Con
LPS
Con+DMSO
LPS+DMSO
Con+TAK242
LPS+TAK242
marker
x
x
x
x
x
x
70kD
55kD
65kD

Fig 5A ECL

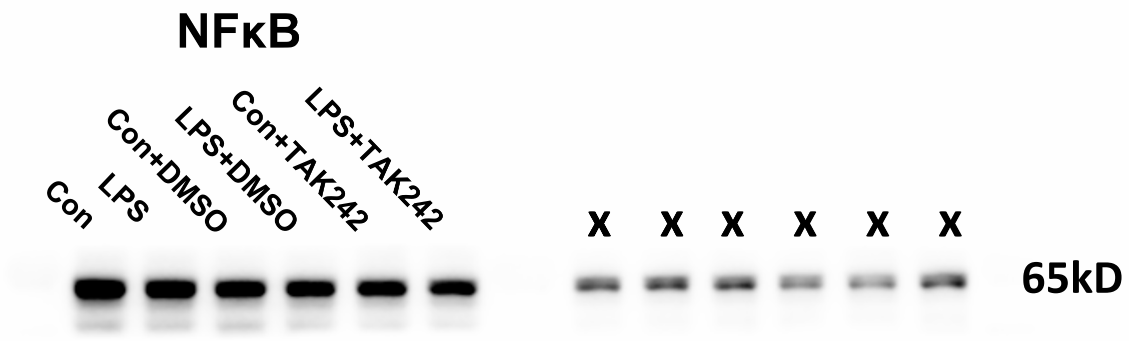
NFκB
Con
LPS
Con+DMSO
LPS+DMSO
Con+TAK242
LPS+TAK242
x
x
x
x
x
x
65kD

Fig 5A   ECL

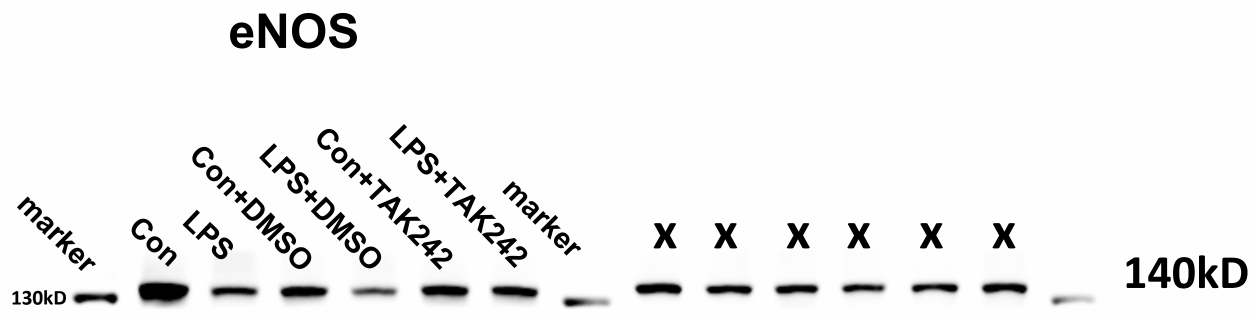

eNOS
marker
Con
LPS
Con+DMSO
LPS+DMSO
Con+TAK242
LPS+TAK242
marker
X
X
X
X
X
X
130kD
140kD

Fig 5A ECL

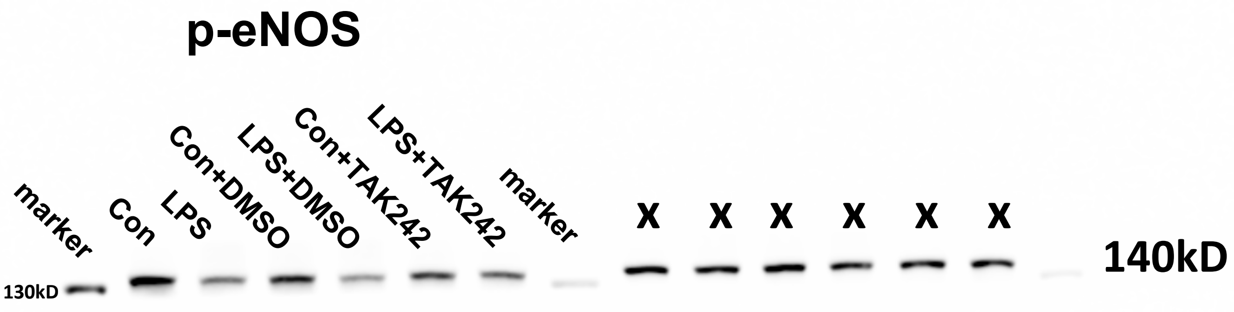
p-eNOS
marker
Con
LPS
Con+DMSO
LPS+DMSO
Con+TAK242
LPS+TAK242
marker
X
X
X
X
X
X
130kD
140kD

Fig 5A ECL

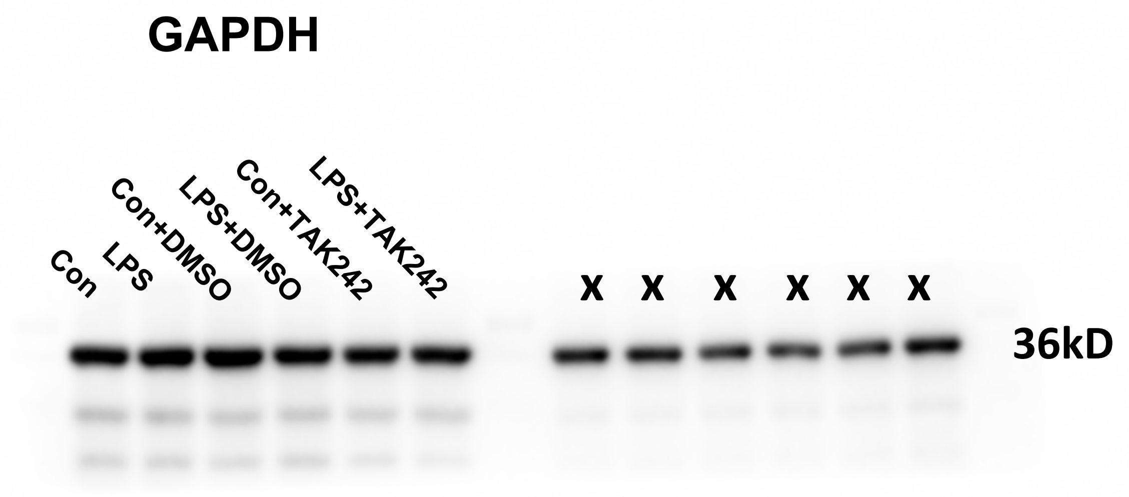
GAPDH
Con
LPS
Con+DMSO
LPS+DMSO
Con+TAK242
LPS+TAK242
x
x
x
x
x
x
36kD

Fig 5D ECL

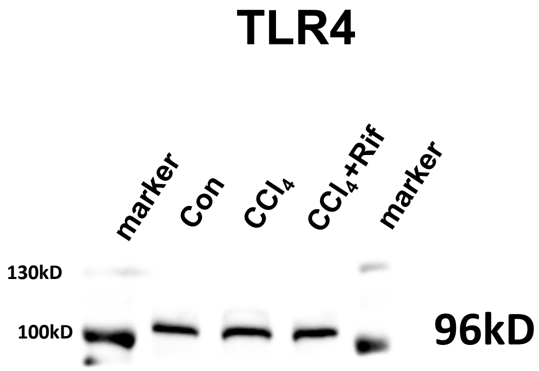

Fig 5D ECL

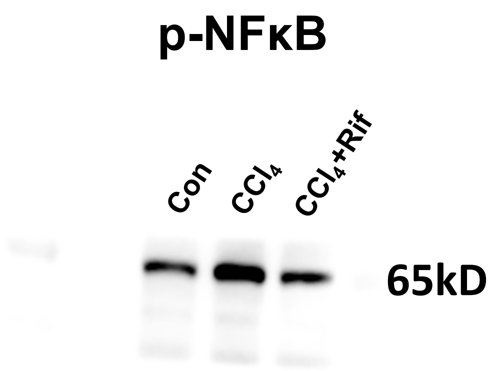

Fig 5D ECL

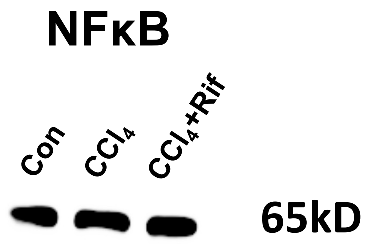

Fig 5D ECL

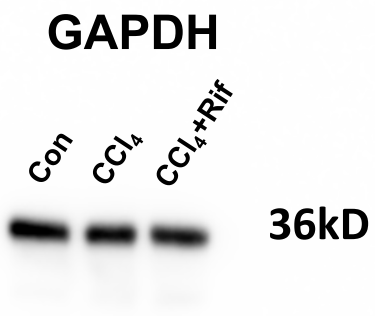

Fig 5D ECL

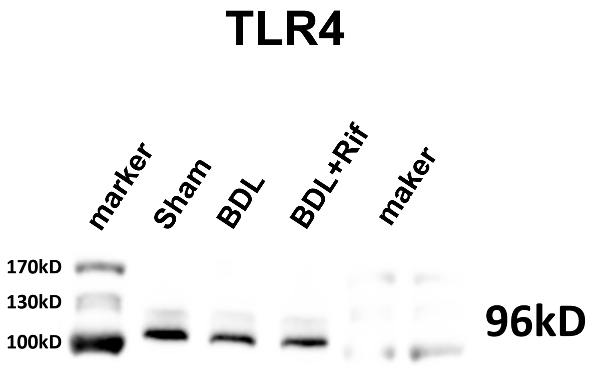

Fig 5D ECL

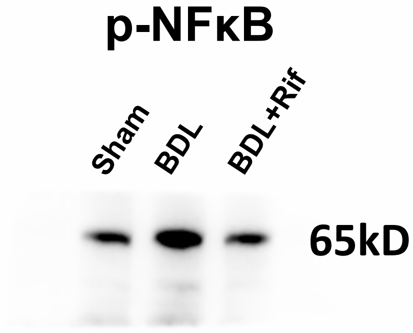

Fig 5D ECL

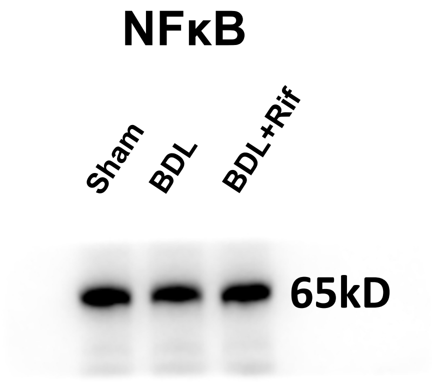

Fig 5D ECL

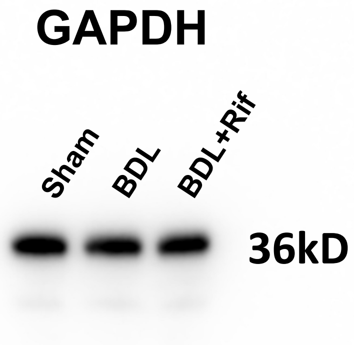

Supplement: S1 Data — (PDF) [file pone.0340664.s002.pdf]
